# Triglycerides, Glucose Metabolism, and Type 2 Diabetes

**DOI:** 10.3390/ijms26209910

**Published:** 2025-10-11

**Authors:** Yutang Wang

**Affiliations:** Discipline of Life Science, Institute of Innovation, Science and Sustainability, Federation University Australia, Ballarat, VIC 3353, Australia; yutang.wang@federation.edu.au

**Keywords:** hypertriglyceridemia, diabetes, metabolic syndrome, cardiometabolic disease, fish oil, fatty acid, inflammation, oxidative stress

## Abstract

Type 2 diabetes is a major global health burden, causing approximately 2 million deaths annually. Recent studies have revealed a strong positive correlation between elevated triglyceride levels and plasma glucose, as well as increased prevalence, incidence, and mortality of type 2 diabetes, suggesting a potential causal link. This review explores the metabolic interconversion between triglycerides and glucose, emphasizing how excess carbohydrate intake leads to ectopic triglyceride accumulation, which in turn enhances hepatic gluconeogenesis. It highlights key signaling pathways through which ectopic triglyceride deposition drives insulin resistance, hyperinsulinemia, β-cell dysfunction and apoptosis, and increased glucose production—central mechanisms in diabetes pathogenesis. Evidence from clinical interventions, such as the reversal of type 2 diabetes through bariatric surgery and dietary energy restriction, supports the hypothesis that ectopic triglyceride accumulation is a driving factor. Furthermore, this review explains why omega-3 fatty acids and niacin, in contrast to fibrates, do not protect against type 2 diabetes, despite lowering triglycerides. Overall, this review emphasizes the contribution of ectopic triglyceride accumulation—driven by obesity, hypertriglyceridemia, excessive consumption of carbohydrates and fats, and physical inactivity—to the onset and progression of type 2 diabetes, offering valuable insights into potential therapeutic strategies.

## 1. Introduction

An abundant food supply and insufficient physical activity in modern society pose significant challenges to cellular metabolism. These lifestyle factors increase the risk of obesity, hypertriglyceridemia, and diabetes [1].

Globally, approximately 589 million people are living with diabetes, with type 2 diabetes accounting for around 90% of all cases [2,3]. The disease affects multiple organs, including the eyes, kidneys, and cardiovascular system, leading to complications such as blindness, kidney failure, heart attacks, strokes, and lower limb amputations [4,5,6]. Diabetes also imposes a substantial economic burden. In the U.S. alone, the annual cost is estimated at $413 billion, comprising $307 billion in direct medical expenses and $106 billion in indirect costs [7]. Furthermore, diabetes is a leading cause of death, responsible for 2 million fatalities worldwide each year [4].

This underscores the urgent need for effective prevention and treatment strategies. Notably, 90% of new type 2 diabetes cases are linked to poor lifestyle choices [8], and lifestyle modifications—such as weight loss and increased physical activity—can reduce the risk by up to 60% [9,10].

Growing evidence indicates that triglycerides play a critical role in the pathogenesis of type 2 diabetes [11,12,13,14,15,16,17,18,19]. Elevated circulating triglyceride levels (≥150 mg/dL) are observed in 40–55% of patients with type 2 diabetes [20,21,22]. High triglyceride levels are positively correlated with plasma glucose [11,12], as well as with the prevalence [11,12,13], incidence [14], and mortality of type 2 diabetes [11,13,15]. Triglyceride-lowering medications such as fibrates have shown protective effects in both animal models [16] and human studies [17,18,19].

This review explores the metabolic relationship between triglycerides and glucose and highlights the interconversion of these two types of molecules, emphasizing how excess carbohydrate intake leads to ectopic triglyceride accumulation, which in turn enhances hepatic gluconeogenesis. It focuses on the signaling pathways through which ectopic triglyceride deposition contributes to insulin resistance, hyperinsulinemia, β-cell dysfunction and apoptosis, and increased hepatic gluconeogenesis—key mechanisms in the development of type 2 diabetes. Evidence from bariatric surgery and dietary energy restriction supports the notion that ectopic triglyceride accumulation is a causal factor. Finally, the review discusses why omega-3 fatty acids and niacin, despite lowering triglycerides, do not confer protection against type 2 diabetes, unlike fibrates.

## 2. Type 2 Diabetes: Diagnosis, Classification, and Risk Factors

### 2.1. Diabetes Diagnosis

Diabetes is diagnosed based on one or more of the following criteria: hemoglobin A1c (HbA1c) ≥ 6.5% (48 mmol/mol), fasting plasma glucose ≥ 126 mg/dL (7.0 mmol/L), 2 h plasma glucose during an oral glucose tolerance test (OGTT) ≥ 200 mg/dL (11.1 mmol/L), or a random plasma glucose ≥ 200 mg/dL (11.1 mmol/L) in patients exhibiting classic symptoms of hyperglycemia or experiencing a hyperglycemic crisis [23,24,25,26]. These diagnostic methods vary in sensitivity. For example, HbA1c ≥ 6.5%, fasting plasma glucose ≥ 126 mg/dL, and 2 h plasma glucose ≥ 200 mg/dL during OGTT can identify approximately 30%, 46%, and 90% of diabetes cases, respectively [27]. Emerging evidence suggests that 1 h plasma glucose during OGTT may be more sensitive in detecting intermediate hyperglycemia than the 2 h measurement [2,28,29]. Additionally, postprandial plasma glucose measured 4–8 h after a meal (non-fasting) may aid in diagnosing diabetes [30,31] and predicting disease outcomes [25,32,33].

### 2.2. Classification of Diabetes

Diabetes is traditionally classified into four categories: type 1 diabetes, type 2 diabetes, gestational diabetes, and specific types caused by other factors (e.g., monogenic syndromes or drug-induced diabetes) [23,34]. Type 1 diabetes results from autoimmune destruction of pancreatic β-cells, typically leading to absolute insulin deficiency. In contrast, type 2 diabetes arises from a non-autoimmune, progressive decline in β-cell insulin secretion, often occurring alongside insulin resistance and metabolic syndrome [34].

### 2.3. Risk Factors for Type 2 Diabetes

Numerous factors contribute to the risk of developing type 2 diabetes [35,36,37,38,39], which can be categorized as modifiable and non-modifiable. Modifiable risk factors include overweight and obesity, physical inactivity [35], unhealthy diets (e.g., high in processed foods, sugary beverages, and saturated fats) [40,41,42], smoking, hypertension [43,44], elevated cholesterol [38,45], high triglycerides [46], and prediabetes [39]. Non-modifiable risk factors include older age (especially over 45 years) [35], family history of type 2 diabetes [35], certain racial and ethnic backgrounds (e.g., African American and Asian American) [35,47], and low birth weight [39]. Additional risk factors include non-alcoholic fatty liver disease, polycystic ovary syndrome, and gestational diabetes [35]. Understanding these risk factors supports effective screening, early detection, and timely intervention [37]. Reducing obesity and increasing physical activity are proven strategies for preventing type 2 diabetes [37].

### 2.4. Characteristics of Type 2 Diabetes

Whole-body glucose homeostasis relies on normal insulin secretion by pancreatic β-cells and adequate tissue sensitivity to insulin for glucose uptake [48]. Skeletal muscle is the primary site of glucose disposal, accounting for approximately 80% of glucose uptake under euglycemic hyperinsulinemic conditions [48].

**Figure 1:**
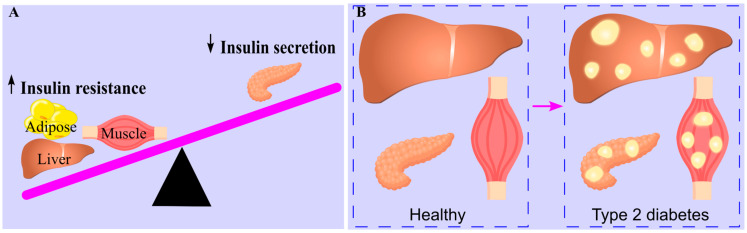
Type 2 diabetes characteristics. This disease is characterized by insulin resistance in the muscle, liver, and adipose tissue and a gradual decrease in insulin secretion by the pancreas (**A**). This disease also displays ectopic triglyceride deposition (**B**). ↓ decrease; ↑, increase.

Insulin resistance and β-cell dysfunction are the key pathophysiological drivers of type 2 diabetes [49,50,51,52], emerging at different stages. Muscle insulin resistance is the earliest detectable abnormality [49,53], prompting the pancreas to increase insulin production to maintain glucose balance [54]. During the prediabetic stage, insulin secretion becomes impaired [50]. Type 2 diabetes develops when insulin production declines alongside increased hepatic gluconeogenesis due to liver insulin resistance [48,50]. Thus, type 2 diabetes is characterized by insulin resistance in muscle and liver, coupled with a progressive loss of pancreatic insulin production (Figure 1).

In established type 2 diabetes, β-cell function continues to deteriorate [50,55], and β-cell mass declines due to increased apoptosis and reduced regeneration [56,57]. This progressive loss contributes to worsening glycemic control [58]. Within 6–10 years of diagnosis, approximately 50% of patients require insulin therapy [59,60].

Ectopic triglyceride deposition is a hallmark of type 2 diabetes. Increased triglyceride accumulation in skeletal muscle [61,62], liver [63], and pancreas [64] has been observed in affected individuals. This deposition is positively associated with insulin resistance [63,65] and diabetes diagnosis [64]. Both lean and obese patients with type 2 diabetes show elevated liver triglyceride content compared to healthy individuals, with obese patients exhibiting even higher levels [63]. These findings suggest that ectopic triglyceride deposition plays a significant role in the pathogenesis of type 2 diabetes [66,67], and the underlying molecular mechanisms will be discussed in detail in this review.

## 3. Triglycerides

### 3.1. Fatty Acids

#### 3.1.1. Fatty Acid Structure

Fatty acids are carboxylic acids with hydrocarbon chains ranging from 4 to 36 carbons long. Some fatty acids are saturated, whereas others are unsaturated, containing one (monounsaturated fatty acids) or more double bonds (polyunsaturated fatty acids). Some common fatty acids are listed in Table 1.

**Figure 2:**
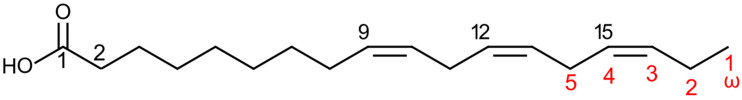
Structure of α-linolenic acid, also known as 18:3 (∆^9,12,15^). It is also an omega-3 fatty acid.

Unbranched fatty acids are named based on their carbon chain length and number of double bonds, separated by a colon (Table 1). For example, the 14-carbon saturated fatty acid myristic acid is denoted as 14:0, while the 18-carbon α-linolenic acid, which contains three double bonds, is written as 18:3. The carboxyl carbon is designated as carbon 1 (C-1), with the adjacent carbon labeled C-2 (Figure 2). Double bond positions are indicated using the delta (∆) symbol, with superscript numbers referring to the lower-numbered carbon in each double bond. Thus, α-linolenic acid is also represented as 18:3(∆^9,12,15^) (Figure 2).

For polyunsaturated fatty acids, an alternative naming system is used. In this system, the carbon farthest from the carboxyl group is referred to as the omega (ω) carbon and designated as C-1, while the carboxyl carbon receives the highest number (Figure 2). Double bond positions are then specified relative to the ω carbon. Fatty acids with a double bond between the third and fourth carbon from the ω end are classified as omega-3 fatty acids, while those with a double bond between the sixth and seventh carbon are classified as omega-6 fatty acids.

Palmitic acid (16:0) is the most common saturated fatty acid in the human body. It can be obtained through the diet or synthesized endogenously, for example, from glucose or other fatty acids [68]. Palmitic acid accounts for approximately 20–30% of total fatty acids in the body [69]. Oleic acid [C18:1 (∆^9^)] is the most abundant unsaturated fatty acid, surpassing even palmitic acid in concentration [70]. It constitutes about 50% of the total triglyceride content in skeletal and adipose tissues and around 35% in the liver [71,72,73,74]. Due to their abundance, palmitic acid and oleic acid are commonly used in studies investigating the functions of fatty acids and triglycerides [75,76,77].

#### 3.1.2. Fatty Acid Biosynthesis

**Figure 3:**
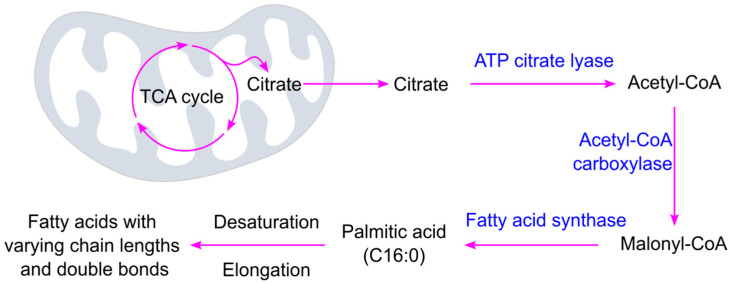
Fatty acid biosynthesis. Citrate, generated from the tricarboxylic acid (TCA) cycle, enters the cytosol from mitochondria and is converted to acetyl-CoA by ATP citrate lyase. The conversion of acetyl-CoA to malonyl-CoA by acetyl-CoA carboxylase is the rate-limiting step in this fatty acid biosynthesis process. Fatty acid synthase catalyzes the formation of the primary end product of palmitic acid, which can be further modified to produce other fatty acids with varying chain lengths and degrees of saturation. Blue text indicates enzymes.

Glucose oxidation produces citrate, which plays a central role in linking cellular energy metabolism with fatty acid synthesis. Citrate is transported from the mitochondria into the cytoplasm, where it is converted into acetyl-CoA by ATP citrate lyase (Figure 3). Acetyl-CoA is then carboxylated to malonyl-CoA by acetyl-CoA carboxylase—this is the rate-limiting step in fatty acid biosynthesis [78]. Fatty acid synthase catalyzes the synthesis of fatty acids from acetyl-CoA and malonyl-CoA in the presence of the reducing agent NADPH (nicotinamide adenine dinucleotide phosphate). The process involves repeated cycles of two-carbon additions (from malonyl-CoA) to a growing fatty acid chain, beginning with a two-carbon acetyl-CoA starter unit. Each cycle includes condensation, reduction, dehydration, and a second reduction step [79]. The primary end product of this process in humans is palmitate, a 16-carbon saturated fatty acid, which can be further modified through elongation and/or desaturation to produce a variety of fatty acids with different chain lengths and degrees of saturation [80].

#### 3.1.3. Fatty Acid Beta-Oxidation (β-Oxidation)

**Figure 4:**
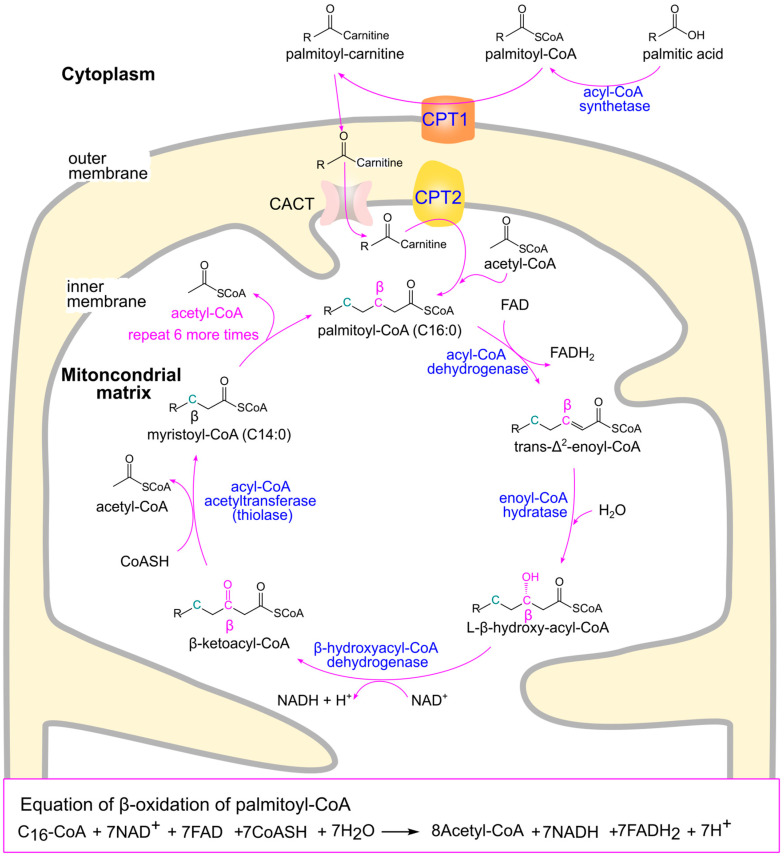
β-oxidation of palmitic acid (16:0). Blue text indicates enzymes. CACT: carnitine-acylcarnitine translocase; CTP1, carnitine palmitoyltransferase 1; CTP2, carnitine palmitoyltransferase 2; FAD, flavin adenine dinucleotide; FADH_2_, reduced flavin adenine dinucleotide; NAD, nicotinamide adenine dinucleotide; NADH, reduced nicotinamide adenine dinucleotide; NAD^+^, oxidized nicotinamide adenine dinucleotide.

β-oxidation is a metabolic process that breaks down fatty acids into acetyl-CoA (Figure 4). This process also generates reduced nicotinamide adenine dinucleotide (NADH) and reduced flavin adenine dinucleotide (FADH_2_), which are subsequently used in other metabolic pathways to produce energy. β-oxidation occurs in the mitochondrial matrix and involves a repetitive sequence of four reactions: oxidation, hydration, a second oxidation, and cleavage (thiolysis), each removing a two-carbon unit from the fatty acid chain [81].

Fatty acids with chain lengths of 12 or fewer carbon atoms can enter mitochondria directly without the need for transporters. However, longer-chain fatty acids (≥14 carbons), which constitute the majority of dietary and endogenous fatty acids, require transport via the carnitine shuttle, which involves three enzymatic steps: (1) Activation: Fatty acids are converted to fatty acyl-CoA by acyl-CoA synthetases at the outer mitochondrial membrane [82]. (2) Transport: Fatty acyl-CoA is converted to fatty acyl-carnitine by carnitine acyltransferase (also known as carnitine palmitoyltransferase 1, CPT1), located on the outer mitochondrial membrane. Fatty acyl-carnitine then diffuses across the intermembrane space and enters the mitochondrial matrix via passive transport through the carnitine-acylcarnitine cotransporter (carnitine-acylcarnitine translocase) in the inner membrane. (3) Reconversion: Inside the matrix, fatty acyl-carnitine is converted back to fatty acyl-CoA by carnitine acyltransferase 2 (CPT2), located on the inner face of the inner mitochondrial membrane [78]. CPT1 is considered the rate-limiting enzyme for the entry of long-chain fatty acids into mitochondria for β-oxidation [83,84].

### 3.2. Glycerol

**Figure 5:**
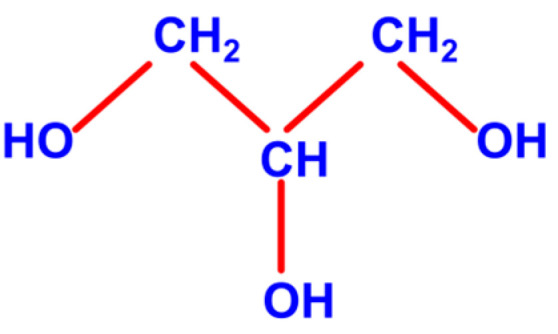
Molecular structure of glycerol.

Glycerol, also known as glycerin, is a colorless, odorless, viscous liquid with a sweet taste. It is a simple triol compound consisting of three carbon atoms (Figure 5) and serves as a backbone for triglyceride synthesis.

### 3.3. Triglyceride Biosynthesis from Glycerol and Fatty Acid

**Figure 6:**
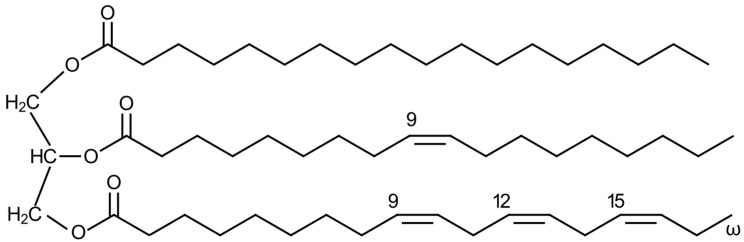
Molecular structure of a triglyceride. This example mixed triglyceride has three different fatty acids attached to the glycerol backbone: they are stearic acid (18:0), oleic acid [18:1 (∆^9^)], and α-linolenic acid [18:3 (∆^9,12,15^)], respectively, from top to bottom.

Triglycerides, also known as triacylglycerols or neutral fats, are composed of three fatty acid molecules esterified to the three hydroxyl groups of a single glycerol molecule (Figure 6). Simple triglycerides contain only one type of fatty acid, whereas most natural triglycerides are mixed, comprising two or three different fatty acids. Triglycerides serve as the primary storage form of fat in the human body [85,86].

**Figure 7:**
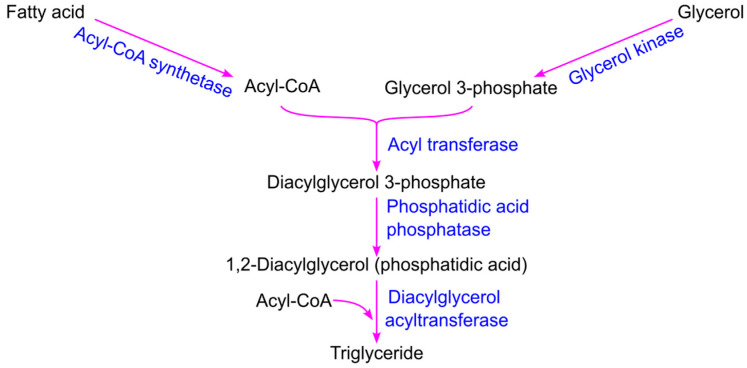
Biosynthesis of triglycerides from glycerol and fatty acids. Blue text indicates enzymes.

Triglyceride biosynthesis begins with the conversion of glycerol to glycerol 3-phosphate by glycerol kinase, while fatty acids are activated to acyl-CoA by acyl-CoA synthetase (Figure 7). Diacylglycerol 3-phosphate—also known as phosphatidic acid or phosphatidate—is formed through the acylation of the two free hydroxyl groups on glycerol 3-phosphate by two acyl-CoA molecules, a reaction catalyzed by acyl transferase. Phosphatidic acid phosphatase then dephosphorylates diacylglycerol 3-phosphate to produce 1,2-diacylglycerol, which subsequently reacts with a third acyl-CoA to form triglycerides [78].

### 3.4. Triglyceride Digestion, Absorption, Delivery, and Storage

**Figure 8:**
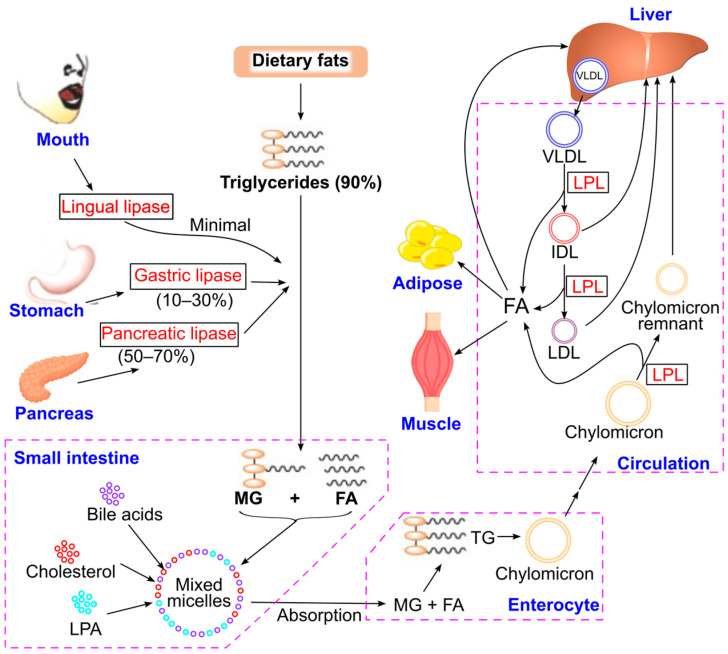
Triglyceride digestion, absorption, delivery, and storage. Dietary fats largely comprise mixed triglycerides (90%), which are hydrolyzed by various lipases for absorption. Pancreatic lipase is the key lipolytic enzyme, responsible for the hydrolysis of 50–70% of dietary fats to monoglycerides (MGs) and fatty acids. The latter two compounds, together with bile salts, cholesterol, and lysophosphatidic acid (LPA), form mixed micelles that are then absorbed into enterocytes. The absorbed MGs and fatty acids will then resynthesize triglycerides to be incorporated into chylomicrons in the enterocytes. Chylomicrons later enter circulation and form fatty acids and chylomicron remnant after their core triglycerides are hydrolyzed by the lipoprotein lipase (LPL). Fatty acids can be taken up by tissue cells, and chylomicron remnants are removed by the liver. The liver forms triglyceride-rich VLDL. In the blood, VLDL triglyceride is hydrolyzed by LPL, generating fatty acids and IDL particles. The IDL particles can be taken up by the liver or form fatty acids and LDL, and the latter will be removed by the liver. FA, fatty acid; IDL, intermediate-density lipoprotein; LDL, low-density lipoprotein; VLDL, very low-density lipoprotein. Adapted with permission from [88]. Copyright year, 2007, with permission from Elsevier.

Approximately 90% of dietary fats consist of mixed triglycerides [87,88]. Before absorption, these molecules undergo hydrolysis in the digestive system by lipases, including lingual, gastric, and pancreatic lipase (Figure 8). Pancreatic lipase, secreted by the pancreas, is the primary enzyme responsible for 50–70% of triglyceride hydrolysis in the intestine [89,90,91]. It exhibits optimal activity at pH 7.0–7.5 and is inactive below pH 5 [89]. Gastric lipase functions best in acidic conditions (pH 3–6) and retains low activity at pH 6–8 [89,92,93]. It plays a supportive role in lipolysis, contributing to 10–30% of triglyceride hydrolysis [87,89,90,91]. In cases of chronic pancreatitis, gastric lipase secretion increases to compensate for reduced pancreatic lipase activity [93,94]. Lingual lipase has minimal activity in adults but is important for hydrolyzing milk triglycerides in infants [95,96].

The resulting monoglycerides and fatty acids, along with bile salts, cholesterol, and lysophosphatidic acids, form mixed micelles that are absorbed by enterocytes [97,98,99]. Inside enterocytes, monoglycerides and fatty acids are re-esterified into triglycerides, which are then packaged into nascent chylomicrons. These chylomicrons are secreted into the lymphatic system and eventually enter the bloodstream.

In capillary beds, triglycerides within chylomicrons are hydrolyzed by lipoprotein lipase, releasing fatty acids that are taken up by adipocytes, myocytes, and hepatocytes for storage or β-oxidation to generate ATP [100,101]. After triglyceride hydrolysis, chylomicrons are converted into chylomicron remnants, which are cleared by the liver.

The liver synthesizes triglycerides by combining glycerol with fatty acids, which are either taken up from plasma or newly synthesized [102]. These triglycerides are incorporated into very-low-density lipoproteins (VLDL), which are rich in triglycerides. In the plasma, VLDL triglycerides are hydrolyzed by lipoprotein lipase, producing fatty acids and intermediate-density lipoprotein (IDL) particles. IDL can be taken up by the liver or further processed by lipoprotein lipase to form fatty acids and low-density lipoprotein (LDL), which is also cleared by the liver. Fatty acids released during these processes can be utilized by various tissues, including adipocytes, myocytes, and hepatocytes. Additionally, hormone-sensitive lipase plays a crucial role in triglyceride hydrolysis within adipose tissue during periods of increased energy demand or fasting [103].

### 3.5. Classification of Triglyceride Levels in Humans

Triglycerides can be obtained from dietary sources through intestinal absorption. They can also be synthesized endogenously by tissue cells such as the liver and muscle, where excess carbohydrates and other nutrients are converted into fatty acids and subsequently into triglycerides—a process known as de novo lipogenesis [104]. Circulating triglyceride levels reflect the dynamic balance between their production (primarily by the liver and intestine) and their clearance (mainly by the liver and muscle) [104].

According to the National Cholesterol Education Program Adult Treatment Panel III (NCEP ATP III) guidelines [102], fasting triglyceride levels in humans are classified into four categories:

- Normal: <150 mg/dL (1.7 mmol/L)
- Borderline high: 150–199 mg/dL (1.8–2.2 mmol/L)
- High: 200–499 mg/dL (2.3–5.6 mmol/L)
- Very high: ≥500 mg/dL (≥5.7 mmol/L)

Approximately 30% of adults have hypertriglyceridemia, defined as a triglyceride level above 150 mg/dL [102,105,106].

## 4. Glucose Metabolism

### 4.1. Common Glucose Metabolism Pathways

**Figure 9:**
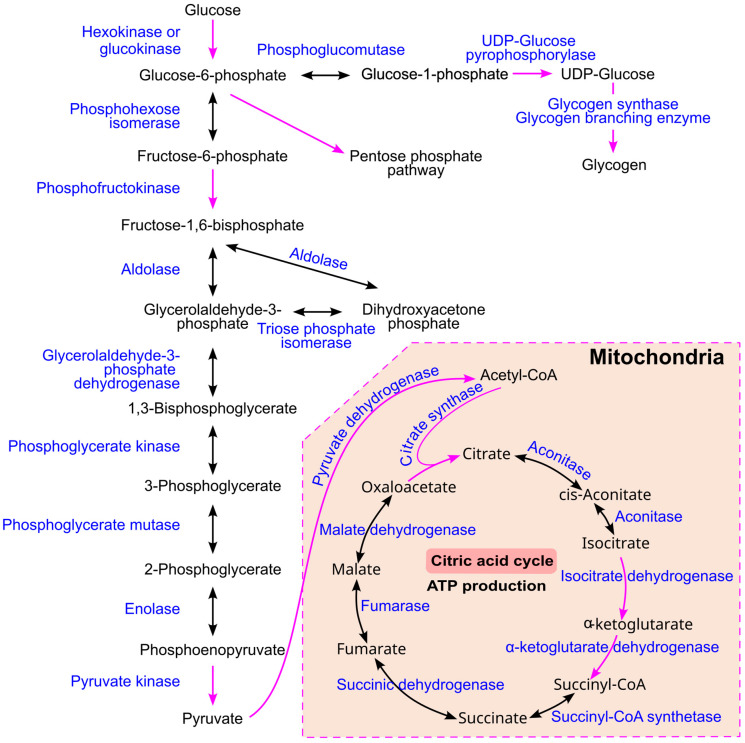
Glucose metabolism. Glucose is converted by hexokinase or glucokinase to glucose-6-phosphate, which can be used for glycogen synthesis or glycolysis to generate pyruvate. Pyruvate enters mitochondria and forms acetyl-CoA by pyruvate dehydrogenase. Acetyl-CoA then enters the citric acid cycle for ATP production following oxidative phosphorylation. Blue text indicates enzymes. Double-headed arrows indicate that these steps are reversible.

Glucose metabolism is well understood and is summarized in Figure 9. Glucose is first converted to glucose-6-phosphate by either hexokinase or glucokinase. This intermediate can be directed toward glycogen synthesis, a process that occurs in virtually all human tissues, with particularly high activity in the liver and muscle [78]. Alternatively, glucose-6-phosphate can enter the glycolytic pathway to produce pyruvate, which is then transported into mitochondria and converted into acetyl-CoA by the enzyme pyruvate dehydrogenase. Acetyl-CoA subsequently enters the citric acid cycle (also known as the Krebs cycle or tricarboxylic acid cycle), followed by oxidative phosphorylation to generate ATP [107].

**Figure 10:**
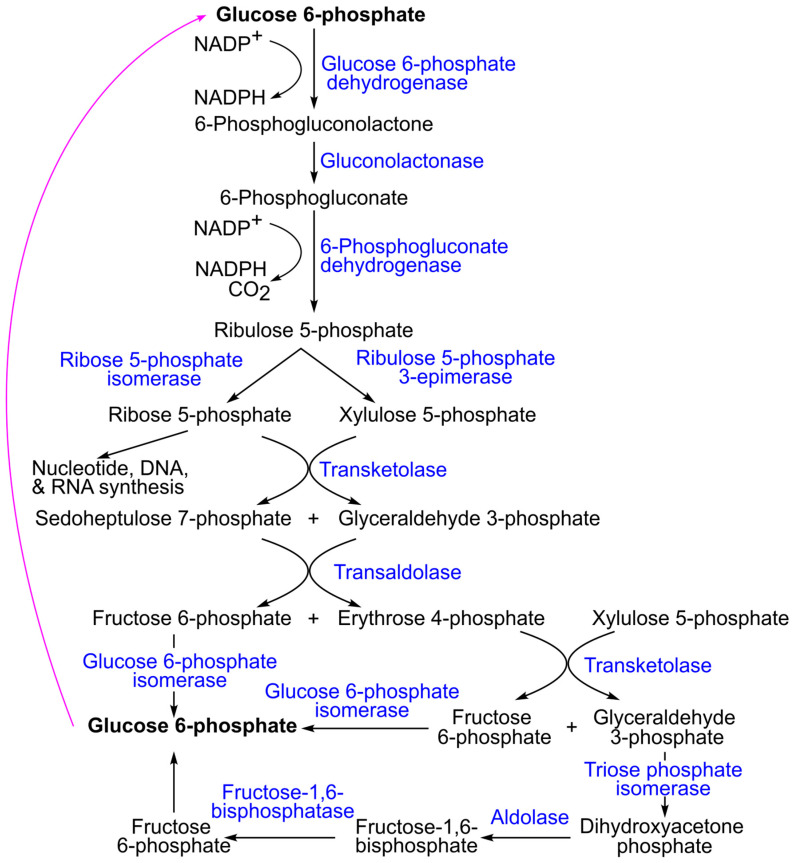
Pentose phosphate pathway. CO_2_, carbon dioxide; NADP^+^, oxidized nicotinamide adenine dinucleotide phosphate; NADPH, reduced nicotinamide adenine dinucleotide phosphate. Blue text indicates enzymes.

In addition, glucose-6-phosphate can be metabolized via the pentose phosphate pathway (also referred to as the phosphogluconate pathway or hexose monophosphate shunt; Figure 10). This pathway is essential for producing NADPH and ribose-5-phosphate, which are required for fatty acid and nucleotide synthesis, respectively [78]. Ribose-5-phosphate can be recycled back into glucose-6-phosphate, allowing the pathway to continue. Several intermediates are shared between the pentose phosphate pathway, glycolysis, and gluconeogenesis. For example, glyceraldehyde 3-phosphate can either proceed through glycolysis to form pyruvate or enter gluconeogenesis to regenerate glucose [78].

### 4.2. Conversion of Glucose to Triglycerides

Intracellular glucose is a vital energy source. It can be stored as glycogen or converted into triglycerides [108]. However, the body can store only a limited amount of glycogen—just a few hundred grams in the liver and muscles—which is just sufficient to meet energy demands for roughly 12 h. When carbohydrate intake exceeds the body’s glycogen storage capacity, the surplus is converted into triglycerides through de novo lipogenesis [78].

**Figure 11:**
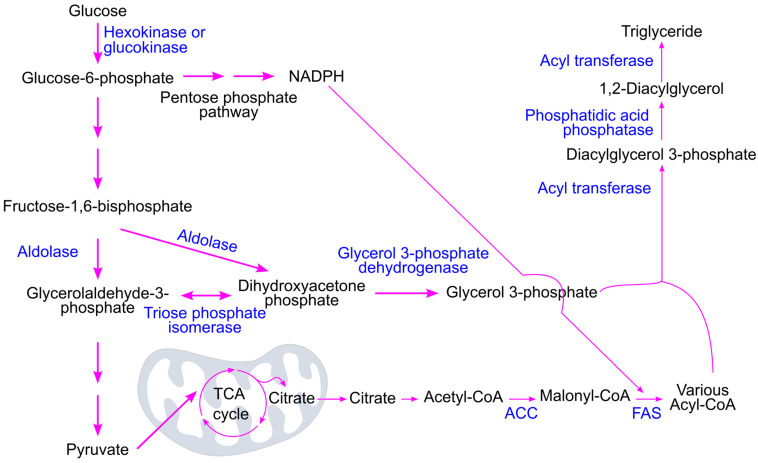
Conversion of glucose to triglycerides. Glucose metabolism facilitates triglyceride formation by producing two precursor molecules (glycerol 3-phosphate and acyl-CoA) as well as NADPH (required for acyl-CoA formation). ACC, acetyl-CoA carboxylase; FAS, fatty acid synthase; NADPH, reduced nicotinamide adenine dinucleotide phosphate; TCA, tricarboxylic acid. Blue text indicates enzymes.

Glucose metabolism supports triglyceride synthesis by generating two key precursors: glycerol 3-phosphate and acyl-CoA (Figure 11). The glycolytic intermediate dihydroxyacetone phosphate (DHAP) is converted into glycerol 3-phosphate by glycerol 3-phosphate dehydrogenase [78,109]. Meanwhile, pyruvate, the end product of glycolysis, enters the mitochondria and fuels the tricarboxylic acid cycle for ATP production. Excess citrate produced in this cycle is transported to the cytosol, where it is converted into acetyl-CoA, a precursor for acyl-CoA formation.

Additionally, glucose metabolism can proceed through the pentose phosphate pathway, which generates nicotinamide adenine dinucleotide phosphate (NADPH)—a crucial reducing agent required for fatty acid synthesis catalyzed by fatty acid synthase [79,110].

## 5. Association of High Triglycerides with Diabetes Epidemiological Indicators

### 5.1. Triglyceride Levels Are Positively Associated with Plasma Glucose Levels

**Figure 12:**
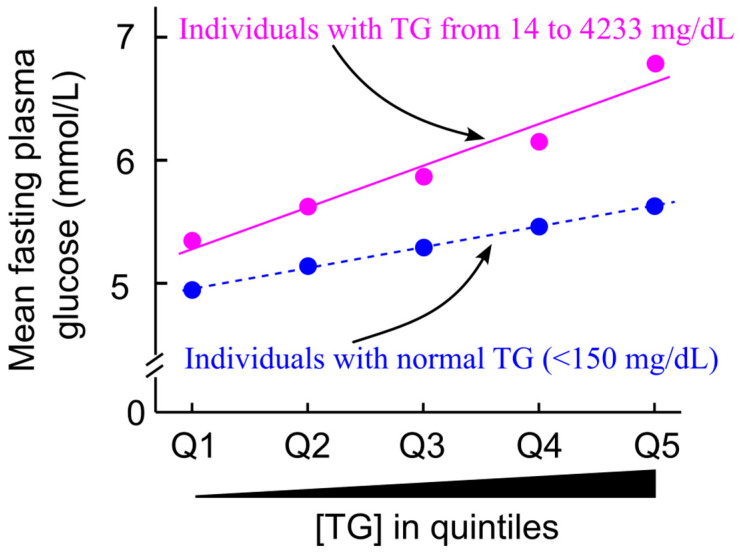
Fasting glucose levels are positively associated with triglyceride levels. This figure is adapted from [11,12], which were published under the terms of the Creative Commons CC BY 4.0 DEED https://creativecommons.org/licenses/by/4.0/ (accessed on 1 July 2025). TG, triglyceride.

Cross-sectional studies have demonstrated a positive correlation between plasma glucose levels and triglycerides across the entire triglyceride spectrum, including within the normal range [11,12] (Figure 12). These findings suggest a close link between triglyceride homeostasis and glucose regulation.

### 5.2. Association of High Triglycerides with Diabetes Prevalence, Incidence, and Mortality

Epidemiological studies have shown that individuals with type 2 diabetes tend to have elevated levels of triglycerides and fatty acids [111,112,113]. Triglyceride levels are positively associated with insulin resistance [11,114] and the prevalence of diabetes [11,12,13,115]. Longitudinal studies further demonstrate that elevated triglyceride levels increase the risk of incidence [14,116,117,118,119,120,121] and mortality associated with diabetes [11,13,122]. Additionally, high triglyceride levels have been associated with an increased risk of cardiovascular mortality among individuals with diabetes [15].

Collectively, at the population level, these findings suggest that elevated triglycerides may contribute to the development and progression of type 2 diabetes. The underlying mechanisms behind this association are discussed in the following sections.

## 6. High Carbohydrate Intake Leads to Ectopic Triglyceride Deposition

Ectopic triglyceride deposition gradually increases over time due to one or more of the following factors: (1) excessive dietary intake of triglycerides and fatty acids, (2) elevated circulating levels of triglycerides and fatty acids, (3) obesity, (4) lack of exercise, or (5) increased triglyceride synthesis from excess glucose [123]. This section focuses on the mechanisms by which excess glucose contributes to ectopic triglyceride accumulation.

**Figure 13:**
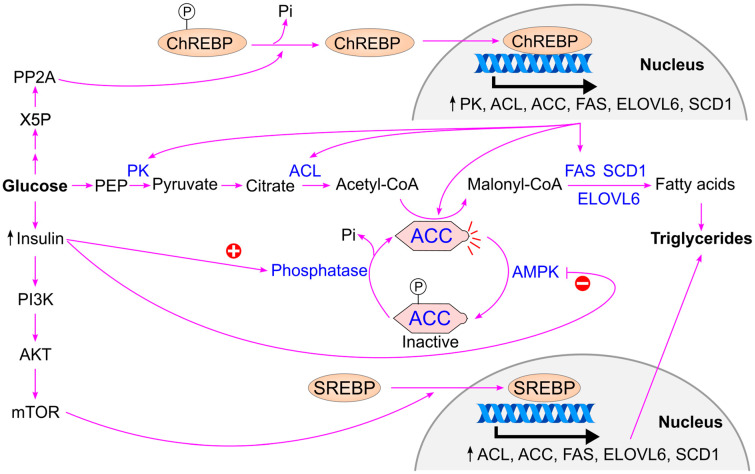
High glucose levels promote triglyceride synthesis. Elevated glucose stimulates insulin secretion, which in turn activates ACC—a key enzyme in fatty acid biosynthesis. Additionally, high glucose enhances the expression of genes involved in triglyceride synthesis by activating two transcription factors, ChREBP and SREBP. ↑, increase; ACC, acetyl-CoA carboxylase; ACL, ATP citrate lyase; AMPK, 5′-AMP-activated protein kinase; ChREBP, carbohydrate response element binding protein; ELOVL6, fatty acid elongase 6; FAS, fatty acid synthase; mTOR, mammalian target of rapamycin; P, phosphate; PEP, phosphoenopyruvate; Pi, inorganic phosphate; PI3K, phosphoinositide 3-kinase; PK, pyruvate kinase; PP2A, protein phosphatase 2A; SCD1, stearoyl-CoA desaturase; SREBP, sterol regulatory element binding protein; X5P, xylulose 5-phosphate. Blue text indicates enzymes.

When blood glucose levels rise, insulin is secreted by the pancreas, activating insulin-dependent protein phosphatase [78,124]. This enzyme dephosphorylates and activates acetyl-CoA carboxylase (ACC), the rate-limiting enzyme in fatty acid biosynthesis. Additionally, insulin inhibits AMP-activated protein kinase (AMPK), thereby maintaining ACC in its active form [125]. Activated ACC catalyzes the conversion of acetyl-CoA to malonyl-CoA, promoting triglyceride synthesis [126] (Figure 13).

Insulin also stimulates the activation of sterol regulatory element-binding protein (SREBP), a transcription factor that upregulates genes involved in fatty acid synthesis, including acetyl-CoA carboxylase, ATP citrate lyase, fatty acid synthase, fatty acid elongase 6, and stearoyl-CoA desaturase [127,128].

High glucose levels further activate carbohydrate response element-binding protein (ChREBP) through increased production of xylulose 5-phosphate via the pentose phosphate pathway. Xylulose 5-phosphate activates protein phosphatase 2A (PP2A), which dephosphorylates ChREBP, enabling its translocation into the nucleus. Once in the nucleus, ChREBP binds to carbohydrate response elements and enhances the expression of genes involved in both glycolysis (e.g., pyruvate kinase) [129,130,131] and fatty acid synthesis (e.g., acetyl-CoA carboxylase, ATP citrate lyase, fatty acid synthase, fatty acid elongase 6, and stearoyl-CoA desaturase) [128,132,133,134].

## 7. Ectopic Triglyceride Deposition Induces Insulin Resistance

### 7.1. Insulin Signaling in Regulating Circulating Glucose

Blood glucose homeostasis is tightly regulated. For instance, when glucose levels rise, the transcription factor carbohydrate-responsive element-binding protein (ChREBP) is activated. In adipose tissue, ChREBP enhances the expression of genes involved in lipogenesis, thereby promoting the conversion of excess glucose into triglycerides [135].

The insulin signaling pathway is the most critical mechanism for maintaining glucose homeostasis. Elevated blood glucose stimulates insulin secretion from the pancreas. Insulin binds to its receptors on target cells, initiating a cascade of molecular events that increase glucose uptake—particularly in muscle and adipose tissue [136]—and suppress hepatic gluconeogenesis. Insulin also promotes the conversion of glucose into triglycerides for energy storage.

**Figure 14:**
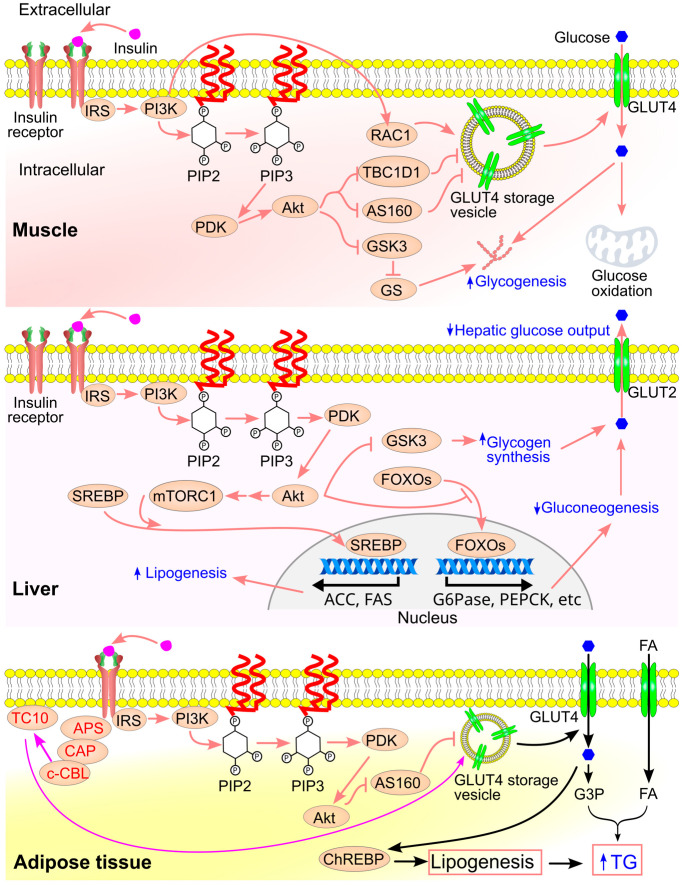
1 Insulin signaling in the muscle, liver, and adipose tissue. Insulin binds to its receptor, leading to IRS phosphorylation. Phosphorylated IRS recruits PI3K, which converts PIP2 to PIP3, and the latter leads to PDK-dependent Akt activation, which in turn leads to increased glucose uptake, oxidation, glycogenesis, conversion of glucose to triglycerides, and decreased gluconeogenesis. Together, these insulin-induced effects in skeletal muscle, liver, and adipose tissue decrease blood glucose. ↑, increase; ↓, decrease; APS, adapter protein with a PH and SH2 domain; AS160, Akt substrate of 160 kDa; CAP, CBL-associated protein; CBL, casitas B-lineage lymphoma; ChREBP, carbohydrate-responsive element binding protein; FA, fatty acid; FAS, fatty acid synthase; FOXO, forkhead box O; G3P, glyceraldehyde 3-phosphate; GS, glycogen synthase; GSK, glycogen synthase kinase; IRS, insulin receptor substrate; PDK, 3-phosphoinositide-dependent protein kinase; PI3K, phosphoinositide 3-kinase; PIP2, phosphatidylinositol 4,5-bisphosphate; PIP3, phosphatidylinositol 3,4,5-triphosphate; RAC1, Ras-related C3 botulinum toxin substrate 1; TBC, the Tre-2/Bub2/Cdc16 domain; TBC1D1, TBC1 domain family member 1; TC10, Rho-related GTP binding protein RhoQ; TG, triglyceride. Blue text indicates the final outcomes of insulin signaling pathways.

Insulin signaling varies across tissues [137] (Figure 14). In skeletal muscle, insulin enhances glucose uptake, oxidation, and glycogen synthesis. In the liver, it inhibits gluconeogenesis while stimulating glycogen synthesis and lipogenesis. In adipose tissue, insulin promotes both glucose uptake and lipogenesis [137].

The pathway begins with insulin binding to the extracellular domain of the insulin receptor, a receptor tyrosine kinase. This triggers autophosphorylation and recruitment of insulin receptor substrates 1 and 2 (IRS1/2), which are phosphorylated on tyrosine residues [138,139]. Phosphorylated IRS proteins recruit phosphoinositide 3-kinase (PI3K), leading to the formation of phosphatidylinositol 3,4,5-triphosphate (PIP3). PIP3 activates 3-phosphoinositide-dependent protein kinase (PDK) [140], which in turn activates Akt, a key kinase that mediates many of insulin’s metabolic effects:

- Akt phosphorylates and inactivates glycogen synthase kinase-3 (GSK3), resulting in activation of glycogen synthase and increased glycogen synthesis [141].
- Akt phosphorylates forkhead box O (FOXO), causing its translocation from the nucleus to the cytoplasm [142], thereby suppressing the expression of gluconeogenic genes in the liver [143,144].
- Akt phosphorylates tuberous sclerosis complex 2 (TSC2) and proline-rich Akt substrate of 40 kDa (PRAS40), leading to activation of mechanistic target of rapamycin complex 1 (mTORC1) [145,146]. mTORC1 promotes cleavage and nuclear translocation of sterol regulatory element-binding protein (SREBP), which upregulates lipogenic gene expression [147,148].

Insulin also stimulates the translocation of GLUT4 to the plasma membrane, resulting in a 10–100-fold increase in glucose uptake [149]. In the absence of insulin, only ~5% of GLUT4 is present on the membrane, whereas insulin stimulation recruits ~40% of total GLUT4 to the cell surface [139,150,151,152]. This translocation is essential for glucose uptake in muscle and adipose tissue and is mediated by several mechanisms:

- Akt phosphorylates TBC1D4/AS160 (TBC1 domain family member 1/Akt substrate of 160 kDa), a Rab GTPase-activating protein, promoting GLUT4 vesicle trafficking to the membrane. In the absence of insulin, AS160 inhibits GLUT4 movement; upon insulin stimulation, AS160 is phosphorylated and inactivated, allowing vesicle translocation and fusion with the membrane [153].
- Akt also phosphorylates and inactivates TBC1D1, another Rab-GAP protein that restricts GLUT4 translocation [154,155].
- PI3K activates RAC1 (Ras-related C3 botulinum toxin substrate 1), a small GTPase that facilitates GLUT4 recruitment to the membrane in muscle cells [156,157].
- The activated insulin receptor binds APS (adapter protein with PH and SH2 domains), which recruits a complex containing c-CBL and c-CBL-associated protein. This leads to c-CBL phosphorylation and activation [158,159]. Activated c-CBL recruits CRK, which activates TC10 (RhoQ), a small GTPase. TC10 interacts with the exocyst tethering complex, enabling docking of GLUT4 vesicles at the cell surface [158,160].

### 7.2. Insulin Resistance

Skeletal muscle plays a central role in glucose clearance under fasting conditions, particularly when muscle glycogen stores are depleted [161]. Glucose taken up by muscle cells is primarily converted into glycogen to replenish these stores [161,162] and excess glucose is converted to triglycerides. Excess glucose is also taken up by adipose tissue and the liver to help maintain circulating glucose homeostasis.

Insulin regulates glucose homeostasis by binding to its receptors on target cells. Disruption of insulin receptor function leads to insulin resistance in various tissues. Interestingly, mice with muscle-specific insulin receptor deficiency maintain normal blood glucose levels [163]. This is due to compensatory glucose uptake by adipose tissue, as these mice exhibit increased insulin-stimulated glucose transport into fat cells [163]. Similarly, mice lacking insulin receptors in adipose tissue [164] or in both muscle and adipose tissues [165] also maintain normal glucose levels, as the liver and pancreas compensate by increasing circulating insulin and glucose uptake [165]. In contrast, mice with liver-specific insulin receptor deficiency develop diabetes [166]. Moreover, mice lacking insulin receptors in both the liver and pancreatic β-cells exhibit severe hyperglycemia and die prematurely at around six weeks of age [166], indicating that insulin resistance in the liver and pancreas critically disrupts glucose homeostasis and contributes to the development of type 2 diabetes [167].

In patients with type 2 diabetes during the postabsorptive state, hepatic glucose production increases modestly by approximately 0.5 mg/kg/min. For a 70 kg individual, this equates to an additional 50 g of glucose released into the circulation over 24 h [62]. Furthermore, basal hepatic glucose production is strongly correlated with fasting hyperglycemia (r = 0.920, *p* < 0.001) [62]. These findings underscore that excessive hepatic glucose output is a major contributor to elevated fasting plasma glucose in type 2 diabetes, despite higher fasting insulin levels compared to healthy individuals. Thus, hepatic insulin resistance is a key pathological feature of type 2 diabetes.

### 7.3. Insulin Resistance Induced by Ectopic Triglyceride Deposition

An increased intake of sugar and fat, along with elevated circulating levels of triglycerides and fatty acids, can lead to greater intramuscular and intrahepatic triglyceride deposition [168,169,170]. Intracellular triglyceride levels in muscle and liver are positively correlated with plasma triglyceride concentrations in humans [171,172]. Notably, intramuscular lipid content is strongly inversely associated with insulin sensitivity—for example, correlation coefficients of r = −0.98 in the tibialis anterior and r = −0.97 in the soleus muscle [168]. Furthermore, a high-fat diet for just three days in humans significantly increases ectopic triglyceride deposition in muscle and induces insulin resistance, even without changes in circulating triglyceride or fatty acid levels [123]. These findings suggest that intracellular triglycerides and fatty acids, rather than their circulating counterparts, play a critical role in the development of insulin resistance.

Elevated intracellular triglyceride levels enhance fatty acid β-oxidation and ATP production [162,173], thereby reducing cellular reliance on glucose oxidation [174,175,176]. More importantly, ectopic triglyceride accumulation impairs the cell’s ability to convert excess glucose into triglycerides, contributing to insulin resistance. Indeed, triglyceride infusion induces insulin resistance in both rodents [177] and humans [178], while systemic inhibition of triglyceride oxidation using etomoxir improves insulin sensitivity in skeletal muscle and adipose tissue [179,180]. High-fat diets have similarly been shown to induce insulin resistance in both animal models [180] and humans [168]. In rats, infusion of triglycerides/heparin to elevate circulating fatty acids leads to whole-body insulin resistance, accompanied by reduced glycogen synthesis, glucose oxidation, and glucose uptake in muscle [181,182]. Likewise, Intralipid infusion in healthy individuals inhibits glucose oxidation [183], decreases glycogen synthesis and content in muscle [184], and reduces ATP production from glucose, impairing glucose storage as glycogen and triglycerides in muscle, liver [108], and adipose tissue [185]. These findings indicate that reduced cellular demand for glucose and impaired conversion of excess glucose to triglycerides are key mechanisms underlying triglyceride-induced insulin resistance.

**Figure 15:**
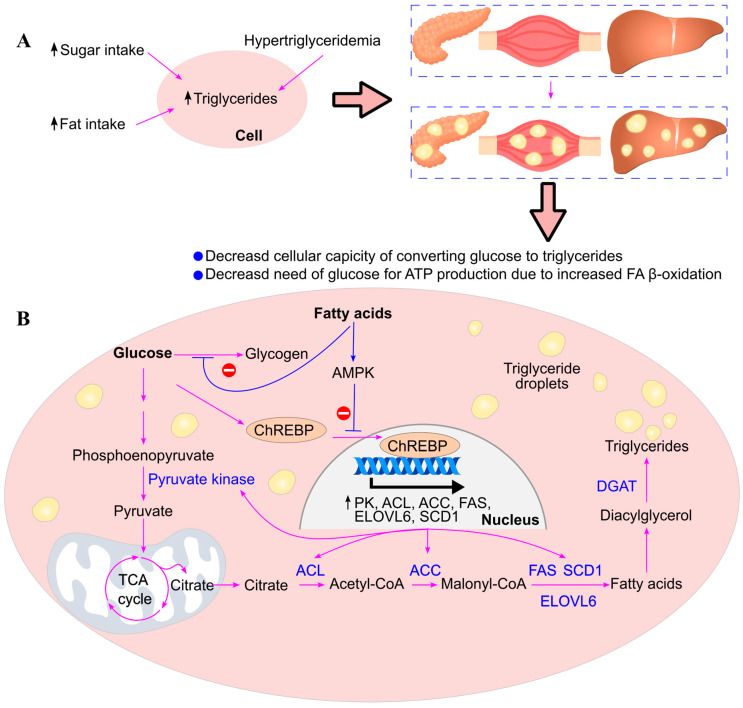
Ectopic triglyceride deposition decreases cellular need for glucose. (**A**), High nutrition intake or hypertriglyceridemia leads to ectopic triglyceride deposition in the muscle, liver and pancreas. (**B**), Ectopic triglyceride deposition decreases the capacity of conversion of glucose to triglycerides. Mechanistically, enhanced intracellular levels of fatty acids inhibit the expression of genes involved in glucose glycolysis and triglyceride synthesis via AMPK-mediated inhibition of ChREBP. In addition, fatty acids inhibit glycogen synthesis. ↑, increase; ACC, acetyl-CoA carboxylase; ACL, ATP citrate lyase; AMPK, 5′-AMP-activated protein kinase; ChREBP, carbohydrate response element binding protein; DAG, diacylglycerol; DGAT, diacylglycerol acyltransferase; ELOVL6, fatty acid elongase 6; FA, fatty acid; FAS, fatty acid synthase; IRS1, insulin receptor substrate-1; PI3K, phosphoinositide 3-kinase; SCD1, stearoyl-CoA desaturase. Blue text indicates enzymes.

ChREBP plays a central role in converting glucose to triglycerides by promoting the expression of genes involved in glycolysis (e.g., pyruvate kinase) and fatty acid synthesis (e.g., fatty acid synthase). Fatty acids activate AMP-activated protein kinase (AMPK), which phosphorylates ChREBP, reducing its DNA-binding capacity and promoting its exclusion from the nucleus [132]. Consequently, fatty acids inhibit ChREBP-mediated expression of glycolytic [129,130,131] and lipogenic genes [128,132,133,134], diminishing the cell’s ability to store excess glucose as triglycerides (Figure 15).

**Figure 16:**
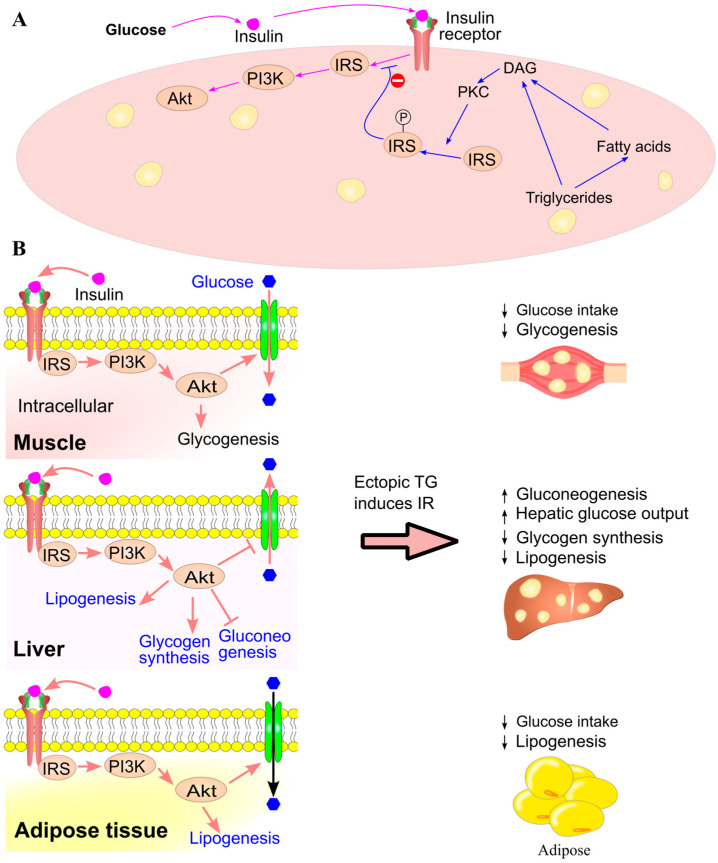
Ectopic triglyceride deposition induces insulin resistance. (**A**), Increased cellular triglyceride and fatty acids increase DAG, which activates PKC. PKC phosphorylates and prevents the activation of IRS by insulin receptor. Thus, triglycerides/fatty acids inhibit insulin signaling and lead to insulin resistance. (**B**), Insulin resistance leads to various responses in different cell types. For example, glucose intake and glycogenosis are decreased in muscle cells, and gluconeogenesis and hepatic glucose output are increased in liver cells, and glucose uptake and lipogenesis are decreased in adipose tissue. ↑, increase; ↓, decrease; DAG, diacylglycerol; IRS1, insulin receptor substrate-1; PI3K, phosphoinositide 3-kinase; PKC, protein kinase.

Elevated intracellular fatty acid levels also increase the biosynthesis of diacylglycerol [186,187], which activates protein kinase C theta (PKCθ) [181,187,188] (Figure 16). PKCθ phosphorylates insulin receptor substrate-1 (IRS1) on serine residues, impairing its tyrosine phosphorylation and downstream activation of the Akt pathway [181,189]. This disruption in insulin signaling leads to insulin resistance and reduced glucose uptake [190,191]. In fact, elevated plasma fatty acids significantly reduce insulin-stimulated whole-body glucose uptake in healthy individuals [192,193], with this defect preceded by a decline in glycogen synthesis in skeletal muscle [192].

Fatty acids also stimulate gluconeogenesis, both in vitro [173] and in vivo [176,194]. Basal plasma fatty acid levels are positively correlated with basal hepatic glucose production [195,196]. In mice, a high-fat diet impairs whole-body glucose disposal, reduces muscle glucose oxidation, and diminishes insulin’s ability to suppress hepatic glucose production [180]. Notably, this impairment is not mediated by β-oxidation, as inhibition of β-oxidation with etomoxir does not reverse the high-fat diet’s effect on hepatic glucose output [180].

## 8. Ectopic Triglyceride Deposition Induces Hyperinsulinemia

### 8.1. Insulin Secretion Signaling Pathway

Pancreatic β-cells increase insulin secretion after meals to maintain glucose homeostasis. Circulating glucose is the primary regulator of β-cell function [136]. Glucose enters β-cells via glucose transporters GLUT1, GLUT2, and GLUT3 [197], where it is metabolized, leading to an increase in intracellular ATP and a decrease in ADP levels [198,199]. Elevated ATP levels reduce the open probability of ATP-sensitive potassium (K_ATP_) channels, while higher ADP levels increase it [200]. Consequently, an increase in the ATP/ADP ratio causes K_ATP_ channels to close, reducing potassium efflux and resulting in membrane depolarization.

**Figure 17:**
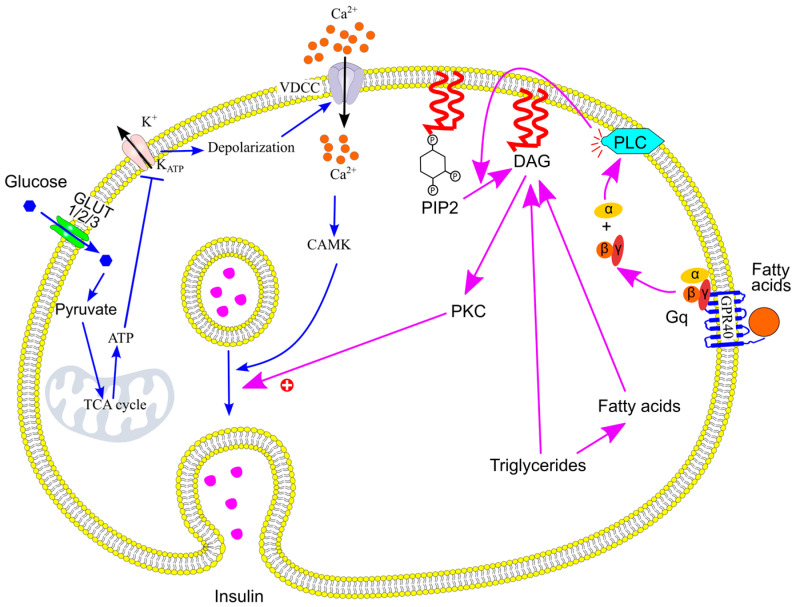
Triglycerides and fatty acids potentiate glucose-stimulated insulin secretion (GSIS). Blue arrows represent GSIS. Purple arrows represent fatty acid-induced potentiation of GSIS. Ca^2+^, calcium ion; CAMK, calcium/calmodulin-dependent protein kinases; DAG, diacylglycerol; GRP40, G-protein-coupled receptor 40; K_ATP_, ATP-sensitive potassium channel; PIP2, phosphatidylinositol 4,5-bisphosphate; PKC, protein kinase C; PLC, phospholipase C; TCA, tricarboxylic acid; VDCC, voltage-dependent calcium channel.

This depolarization activates voltage-dependent calcium channels, allowing calcium ions (Ca^2+^) to enter the cell. The rise in intracellular calcium concentration triggers the fusion of insulin-containing granules with the plasma membrane, leading to insulin release via exocytosis [199,201] (Figure 17).

### 8.2. Acute Increase in Triglycerides and Fatty Acids Potentiate Glucose-Stimulated Insulin Secretion

Triglycerides and fatty acids do not directly stimulate insulin secretion. This is supported by several observations: (1) acute increases in plasma triglycerides following consumption of a fat load (e.g., emulsified corn oil or butter fat) elevate plasma triglyceride and fatty acid levels but do not affect plasma insulin levels in healthy individuals [202,203,204]; (2) co-administration of a fat meal and heparin, which raises plasma fatty acids, does not alter insulin levels [205]; and (3) intravenous infusion of Intralipid and heparin for 48 h also fails to change plasma insulin levels in healthy individuals [206].

However, triglycerides and fatty acids can potentiate glucose-stimulated insulin secretion (GSIS) in the presence of elevated glucose. For example, Pelkonen et al. studied 18 healthy volunteers who consumed 60 g of butter fat after fasting, resulting in a twofold increase in plasma triglycerides [203]. Three hours later, participants received a 25 g intravenous glucose load over two minutes, and GSIS was significantly enhanced. This finding has been confirmed in other studies involving both humans [206] and animal models [204].

Mechanistically, palmitate and other fatty acids with chain lengths ≥12 carbons can bind to and activate G-protein-coupled receptor 40 (GPR40) [207,208] (Figure 17). This activation triggers the Gq–phospholipase C pathway, leading to the production of diacylglycerol (DAG). DAG activates protein kinase C (PKC), which enhances insulin secretion by modulating several downstream targets [209,210,211]. For instance, PKC reorganizes the cortical actin network, facilitating the movement of insulin-containing granules to the plasma membrane [212]. PKC also phosphorylates and activates proteins involved in exocytosis, such as munc18, SNAP25, and synaptotagmin, thereby promoting insulin release [212]. Importantly, PKC activation alone does not trigger insulin secretion [212,213], consistent with the observation that triglycerides and fatty acids do not directly stimulate insulin release [202,203,204].

It is also noteworthy that glycerol does not potentiate GSIS in healthy individuals [203]. Moreover, intracellular triglycerides and fatty acids—rather than their extracellular counterparts—play a critical role in insulin secretion. For example, high-fat diet feeding, which increases intracellular lipid accumulation, can elevate circulating insulin levels without affecting plasma fatty acid concentrations [214].

### 8.3. Ectopic Triglyceride Deposition Promotes Adaptive β-Cell Proliferation as a Response to Insulin Resistance

Ectopic triglyceride deposition contributes to the development of insulin resistance. To maintain glucose homeostasis, the body compensates by increasing insulin secretion in response to prolonged postprandial glucose elevation. Triglycerides and fatty acids can enhance GSIS, temporarily promoting glucose uptake and its conversion into triglycerides for storage [215].

**Figure 18:**
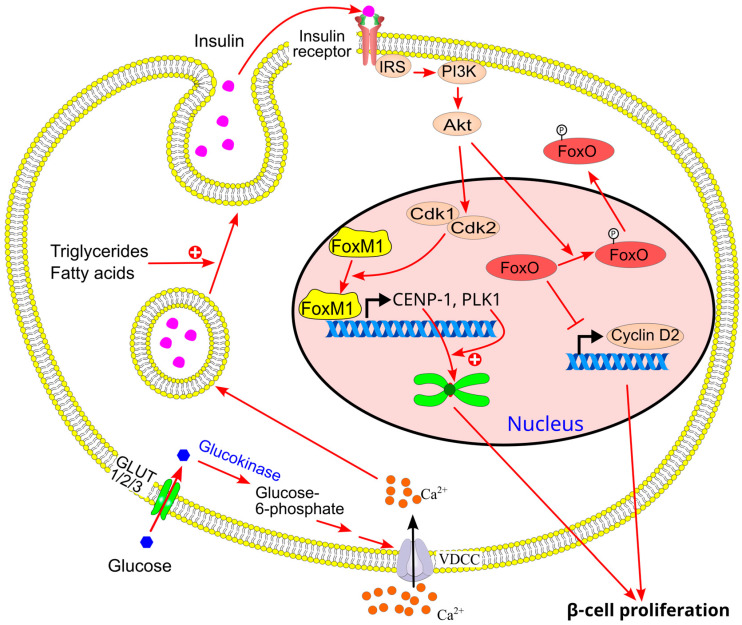
Ectopic triglyceride deposition promotes adaptive β-cell proliferation as a compensatory response to insulin resistance via activation of insulin signaling. Akt, Ak strain transforming, also known as protein kinase B; Cdk, cyclin-dependent kinase; CENP-A, centromere protein A; FoxM1, forkhead box M1; FoxO1, forkhead box O1; IRS, insulin receptor substrate; PI3K, phosphoinositide 3-kinase; PLK1, polo-like kinase-1; VDCC, voltage-dependent calcium channel.

However, over time, triglyceride accumulation in organs such as the liver and muscle increases, exacerbated by hypertriglyceridemia resulting from the conversion of fatty acids into triglycerides within these tissues. As a result, insulin resistance progressively worsens, and the compensatory increase in GSIS becomes insufficient. To counteract this, β-cell proliferation is required to expand β-cell mass and sustain insulin production [209,216,217] (Figure 18). Supporting this, studies have shown that obesity increases relative β-cell volume in humans through enhanced β-cell proliferation [56].

In mice, prolonged high-fat diet (HFD) feeding induces adaptive β-cell proliferation in response to chronic insulin resistance [166,214,218]. These mice exhibit insulin resistance and hyperinsulinemia without developing diabetes, due to compensatory β-cell expansion. HFD-induced β-cell proliferation depends on insulin signaling, as HFD increases the expression of insulin receptor and IRS2 genes in pancreatic islets [216]. Conversely, mice with β-cell-specific deficiencies in the insulin receptor or IRS2 show impaired β-cell proliferation in response to HFD [166,216,219,220].

Glucose metabolism is also essential for this adaptive response. Mice with β-cell-specific glucokinase deficiency fail to exhibit HFD-induced β-cell proliferation [216]. In glucokinase haploinsufficient mice, this defect can be rescued by overexpressing IRS2 in β-cells [216,219]. These findings suggest that: (1) glucose metabolism via glucokinase and insulin signaling via insulin receptor/IRS2 are critical for adaptive β-cell proliferation in response to HFD; and (2) insulin signaling activation occurs downstream of glucose metabolism in this process. Thus, triglyceride-induced β-cell proliferation may be mediated through enhanced GSIS and subsequent activation of insulin signaling via autocrine or paracrine mechanisms [166].

HFD-induced activation of insulin signaling in promoting β-cell proliferation involves forkhead box transcription factors, particularly FoxM1 and FoxO1. Insulin receptor activation enhances FoxM1 DNA-binding activity, regulating the expression of centromere protein A (CENP-A) and polo-like kinase 1 (PLK1) via modulation of cyclin-dependent kinases 1 and 2. PLK1 facilitates CENP-A deposition at the centromere, promoting mitosis [221]. Mice with β-cell-specific CENP-A knockout develop diabetes, accompanied by reduced GSIS and impaired β-cell proliferation under HFD conditions [221]. HFD also induces FoxO1 phosphorylation, leading to its nuclear export and increased expression of cyclin D2, which promotes β-cell proliferation [166,216,219,222].

### 8.4. Ectopic Triglyceride Deposition Leads to Hyperinsulinemia

Ectopic triglyceride deposition contributes to insulin resistance by reducing both the cellular capacity to convert glucose into triglycerides and the cellular demand for glucose as a substrate for ATP production. To maintain glucose homeostasis, the body compensates by increasing insulin secretion to counteract prolonged postprandial hyperglycemia. This compensation involves enhanced GSIS and stimulation of β-cell proliferation. Over time, ectopic triglyceride deposition leads to hyperinsulinemia before the establishment of type 2 diabetes [223,224].

## 9. Ectopic Triglyceride Deposition Impairs β-Cell Function over Time

### 9.1. Chronic Exposure to Fatty Acids Impairs GSIS

Acute exposure to fatty acids is known to enhance GSIS in pancreatic β-cells [206,225]. However, chronic exposure to fatty acids or hyperlipidemia can impair β-cell function—a phenomenon referred to as lipotoxicity [226]. For example, prolonged exposure to palmitic acid in cultured rat pancreatic islets reduces GSIS [75,77,227]. Similarly, 48 h intravenous infusion of fatty acids (e.g., oleate or Intralipid combined with heparin) inhibits GSIS in vivo in rats [228,229]. In mice, high-fat diet feeding decreases GSIS despite an increase in β-cell mass [214,216]. This impaired GSIS is also observed in humans. Carpentier et al. [206] reported that a rapid 90 min infusion of fatty acids increased GSIS in healthy subjects, but this effect disappeared after 48 h of continuous infusion.

Boden et al. [230] also investigated the effects of a 48 h infusion of triglycerides (Liposyn) and heparin in healthy volunteers. Using a hyperglycemic clamp (glucose ~8.6 mmol/L), they found that insulin secretion increased during the 25–48 h period. However, this study is still considered short-term, as it did not assess insulin-stimulated secretion after the infusion ended. Additionally, participants were fasting throughout the 48 h study, with glucose infused to maintain the clamp. Subjects also underwent an overnight fast before the experiment and frequent blood sampling, including during the night, which may not reflect typical β-cell responses under normal daily conditions.

The inhibition of GSIS by chronic exposure to fatty acids and triglycerides is mediated by increased β-oxidation and reduced glucose metabolism. GSIS can be partially restored by inhibiting β-oxidation using etomoxir (a carnitine palmitoyltransferase I inhibitor) [231,232] or bromopalmitic acid [76]. High-fat-diet-induced reductions in GSIS are also associated with decreased glucose oxidation [216]. These findings suggest that ectopic triglyceride deposition reduces β-cell glucose demand due to: (1) impaired capacity to convert glucose into triglycerides, and (2) increased fatty acid β-oxidation, which generates ATP and reduces the need for glucose-derived energy. Supporting this, long-term fatty acid exposure downregulates the expression of GLUT2 and glucokinase in β-cells [76]. Mechanistically, chronic fatty acid exposure suppresses the transcription factor islet/duodenum homeobox-1 (IDX-1) via β-oxidation [76]. IDX-1 enhances GLUT2 and glucokinase expression, and its inhibition leads to impaired glucose sensing in β-cells. Consequently, prolonged elevation of intracellular triglycerides and fatty acids blunts GSIS [76].

### 9.2. Inhibition of Fatty Acids on GSIS Is Reversible

Inhibition of GSIS by prolonged treatment with fatty acids (48 h) is reversible, as it has been shown that 24 h after the removal of fatty acids, GSIS was restored to normal in isolated rat pancreatic islets [75].

### 9.3. Chronic Ectopic Triglyceride Deposition in β-Cells Inhibits Glucose-Induced Increase in Insulin Gene Expression

**Figure 19:**
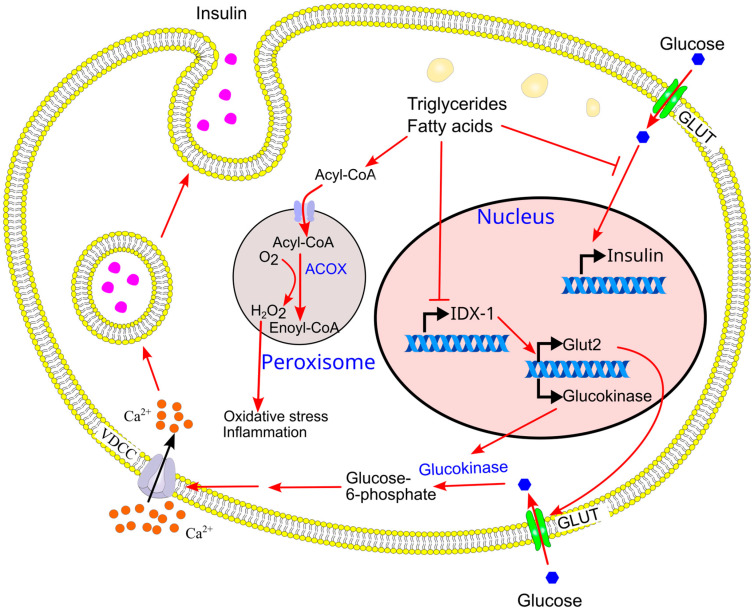
Ectopic triglyceride deposition impairs glucose-stimulated insulin secretion (GSIS) by disrupting key β-cell functions. Intracellular triglyceride accumulation reduces the β-cell’s ability to convert glucose into triglycerides, thereby diminishing glucose uptake and inhibiting the glucose-induced upregulation of insulin gene expression. Additionally, triglyceride buildup suppresses the mRNA expression of the transcription factor IDX-1, leading to reduced expression of glucose transporter 2 (GLUT2) and glucokinase—both essential components of the insulin secretion pathway. Furthermore, enhanced fatty acid β-oxidation within peroxisomes increases hydrogen peroxide (H_2_O_2_) production, contributing to oxidative stress and inflammation. ACOX, acyl-CoA oxidase; GLUT, glucose transporter; IDX-1, islet/duodenum homeobox-1; VDCC, voltage-dependent calcium channel.

Briaud et al. demonstrated that prolonged exposure of isolated rat islets to high glucose concentrations (16.7 mM for 72 h) significantly increased insulin gene expression. However, this glucose-induced upregulation was inhibited when palmitate (0.5 mM) was co-administered over the same duration [123] (Figure 19). Similar inhibitory effects were observed in the β-cell line HIT-T15 [123]. Notably, the suppressive effect of palmitate was dependent on the presence of high glucose; palmitate did not reduce insulin mRNA levels in isolated rat islets cultured under low glucose conditions (2.8 mM) [123]. This glucose-dependent inhibition was further confirmed by Ritz-Laser et al. [233]. Consistently, chronic palmitate treatment reduced insulin content in cultured rat pancreatic islets only in the presence of high glucose [76,77]. These findings suggest that fatty acids do not directly suppress insulin gene expression, but rather interfere with glucose-induced transcriptional activation.

Briaud et al. also reported that excess glucose is converted into triglycerides within pancreatic islets [123]. In HIT-T15 cells exposed to glucose (11.1 mM) for three days, palmitate was incorporated into triglycerides in a dose-dependent manner. Importantly, intracellular triglyceride accumulation was inversely correlated with insulin mRNA levels [123]. Supporting this, the inhibitory effect of palmitate on insulin mRNA expression was abolished when palmitate was replaced with palmitate methyl ester—a compound that cannot be converted into fatty acyl-CoA for triglyceride synthesis [123,233]. These observations suggest that ectopic triglyceride deposition is required for the suppression of glucose-induced insulin gene expression.

Further evidence shows that chronic palmitate exposure impairs glucose-stimulated insulin gene expression [76,77], accompanied by downregulation of key glucose-sensing genes, including glucokinase and the glucose transporter GLUT2, in rat pancreatic islets [76]. The inhibitory effect of palmitate was dependent on its mitochondrial oxidation, as it was prevented by bromopalmitic acid, an inhibitor of carnitine palmitoyltransferase I [76]. These results imply that ectopic lipid accumulation compromises β-cell metabolic flexibility, thereby reducing insulin mRNA levels.

Mechanistically, prolonged fatty acid exposure suppresses insulin gene expression by downregulating the transcription factor IDX-1 (islet/duodenum homeobox-1, also known as Pancreas duodenum homeobox-1 or PDX-1 [234]), which is essential for insulin gene transcription in rat pancreatic islets [76]. Thus, long-term treatment with fatty acids impairs insulin gene expression through transcriptional repression mediated by reduced IDX-1 levels.

### 9.4. High Triglycerides Cause Pancreatitis

Elevated plasma triglyceride levels are a recognized risk factor for pancreatitis [235]. Conversely, pharmacological reduction in triglycerides using agents such as fenofibrate has been shown to lower the incidence of pancreatitis [236]. Accordingly, the American Heart Association recommends triglyceride-lowering interventions in individuals with circulating triglyceride levels ≥ 500 mg/dL to prevent pancreatitis [235]. Indeed, fatty acid β-oxidation in peroxisomes generates hydrogen peroxide, which is catalyzed by acyl-CoA oxidase [237,238]. Rapid hydrogen peroxide accumulation, especially when combined with superoxide radicals to form highly reactive hydroxyl radicals [239], can overwhelm antioxidant defenses, leading to oxidative stress and inflammation [240]. Therefore, it is plausible that hypertriglyceridemia may impair insulin secretion indirectly through the development of pancreatitis (Figure 19).

## 10. Long-Term Ectopic Triglyceride Deposition in the Liver Enhances Gluconeogenesis

### 10.1. Glucoseogenesis from Triglycerides

**Figure 20:**
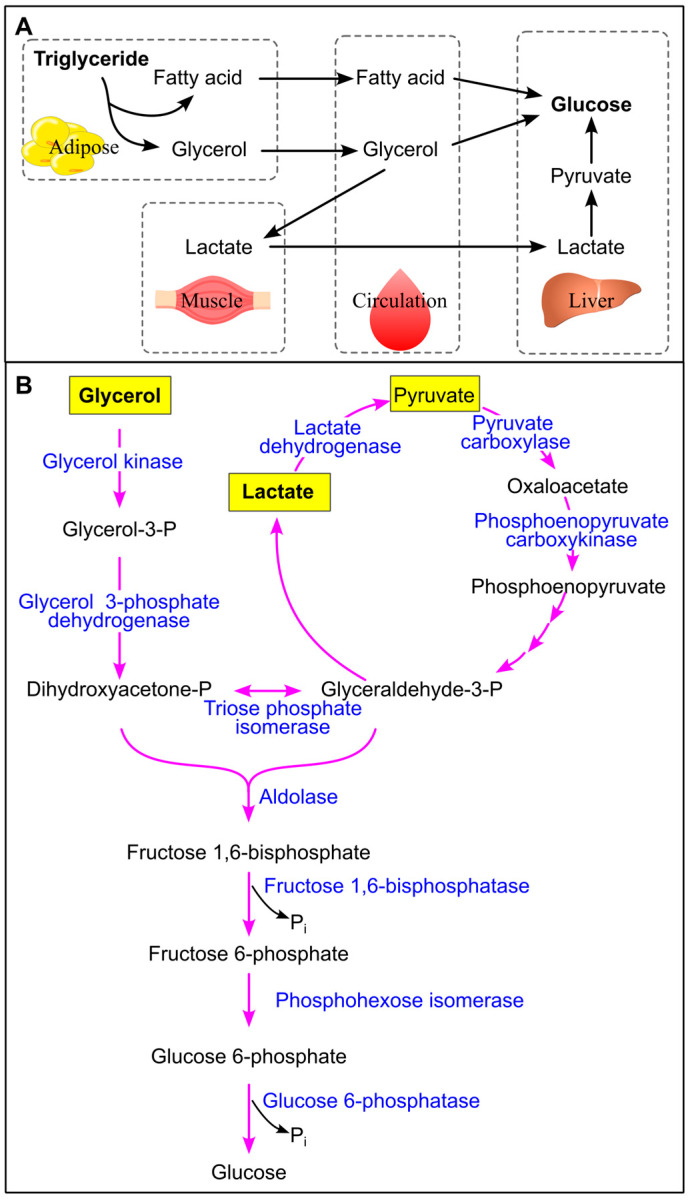
Gluconeogenesis from triglycerides. (**A**), Glycerol acts as a precursor for gluconeogenesis in the liver or is converted in the muscle to lactate as another precursor of gluconeogenesis. In addition, fatty acids can be converted to glucose. (**B**), Detailed molecular pathways of gluconeogenesis from glycerol, lactate, and pyruvate. P, phosphate; P_i_, inorganic phosphate. Blue text indicates enzymes. Molecules with a yellow highlight are common precursors of gluconeogenesis.

Glycerol, a byproduct of triglyceride breakdown, can be converted into glucose in humans, primarily within the liver [241,242,243,244] (Figure 20). Additionally, glycerol may be metabolized into lactate in muscle tissue [245], which subsequently serves as a substrate for hepatic gluconeogenesis [242]. In the liver, lactate is further converted into pyruvate, another key precursor for gluconeogenesis [246] (Figure 20). Fatty acids can be converted into glucose in mammals, as ^14^C-labeled fatty acids can be found to be incorporated into glucose molecules [247,248]. However, the precise pathway by which fatty acids contribute to gluconeogenesis in humans remains to be fully elucidated [249].

### 10.2. Long-Term Ectopic Triglyceride Deposition in the Liver Enhances Gluconeogenesis

Chronic overconsumption of carbohydrates and fats or hypertriglyceridemia leads to ectopic triglyceride accumulation in the liver. In response, the liver activates compensatory mechanisms to restore lipid homeostasis and limit further fat deposition. These include increased secretion of very-low-density lipoproteins and enhanced gluconeogenesis, whereby triglyceride-derived substrates are converted into glucose. As a result, persistent ectopic triglyceride accumulation promotes hepatic gluconeogenesis. Furthermore, triglyceride-induced hepatic insulin resistance diminishes insulin’s inhibitory effect on gluconeogenesis, further amplifying glucose production.

## 11. Severe and Long-Term Ectopic Triglyceride Deposition Induces β-Cell Apoptosis

### 11.1. Ceramide Formation and β-Cell Apoptosis

**Figure 21:**
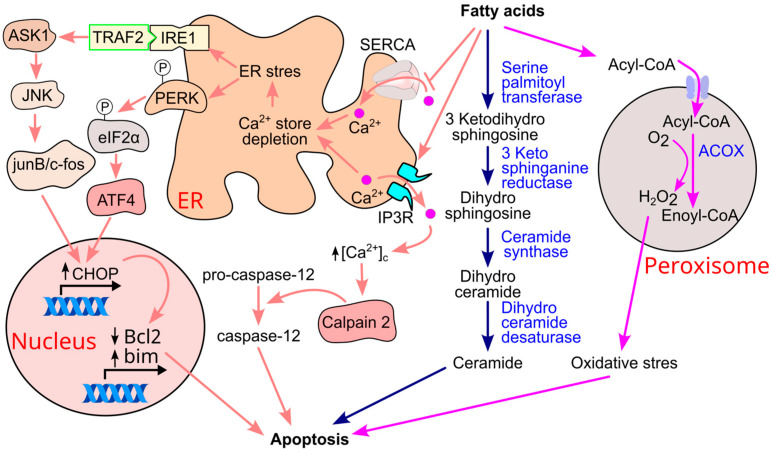
Severe and long-term ectopic triglyceride deposition leads to β-cell apoptosis. Ectopic triglyceride deposition leads to a persistent increase in intracellular fatty acids, which can result in β-cell apoptosis via enhanced oxidative stress, ER stress, and ceramide formation. Fatty acids cause ER stress through Ca^2+^ store depletion, which can lead to activation of ER stress transducers IRE1 and PERK. IRE1 activates JNK and junB and c-fos. Activated PERK phosphorylates eIF2α, which induces expression of transcription factor ATF4. Both junB/c-fos dimer and ATF4 induce CHOP expression and subsequent decrease in anti-apoptotic Bcl2 and increase in proapoptotic bim, increasing β-cell apoptosis. In addition, ER Ca^2+^ store depletion leads to an increase in cytosolic Ca^2+^, which activates calpain 2 and subsequently activation of caspase-12, eventually apoptosis. In addition, a persistent increase in intracellular fatty acids leads to the formation of ceremide to promote apoptosis. Moreover, a persistent increase in intracellular fatty acids results in an increase in fatty acid β-oxidation in the peroxisome which generates excess H_2_O_2_, leading to oxidative stress and inflammation, eventually apoptosis. ↑, increase; ↓, decrease; ACOX, acyl-CoA oxidase; AFT4, activating transcription factor -4; ASK1, apoptosis signal-regulating Kinase 1; Bcl2, B-cell lymphoma 2; bim, Bcl-2-interacting mediator of cell death; Ca^2+^, calcium; [Ca^2+^]_c_, cytosolic calcium concentration; CHOP, C/EBP homologous protein; c-fos, cellular Finkel-Biskis-Jinkins osteosarcoma viral oncogene homolog; eIF2α, eukaryotic translation initiation factor 2α; ER, endoplasmic reticulum; IP_3_R, 1,4,5 trisphosphate receptor; IRE1, inositol-requiring enzyme-1; JNK, c-JunN-terminal kinase; PERK, protein kinase RNA–like ER kinase; SERCA, sarco/endoplasmic reticulum calcium ATPase; TRAF2, TNF receptor-associated factor 2. Blue text indicates enzymes.

Saturated fatty acids such as palmitate, myristate, and stearate can be metabolized into ceramide, a bioactive lipid known to induce β-cell apoptosis [250,251] (Figure 21). For instance, treatment with palmitic acid (0.5 mmol/L) for four days in the presence of high glucose (11.1 mmol/L) significantly increased β-cell apoptosis, an effect mediated by ceramide formation. Notably, inhibition of ceramide synthase reversed this apoptotic response, confirming the role of ceramide in palmitate-induced β-cell death [77]. Mechanistically, palmitic acid upregulates the expression of serine palmitoyltransferase in isolated rat islets [252], the enzyme responsible for catalyzing the first step in ceramide biosynthesis [253]. In genetically diabetic obese fa/fa Zucker rats, increased pancreatic apoptosis was associated with elevated serine palmitoyltransferase expression, and pharmacological inhibition of this enzyme reduced apoptosis in vivo [252].

### 11.2. Fatty Acid-Induced ER Stress and Apoptosis

Elevated levels of fatty acids can also trigger endoplasmic reticulum (ER) stress, a cellular response to the accumulation of misfolded proteins, which can lead to apoptosis [254,255,256] (Figure 21). Fatty acids induce ER stress by depleting ER calcium stores—both by promoting calcium release via the inositol 1,4,5-trisphosphate (IP3) receptor [257] and by inhibiting calcium reuptake through suppression of the sarco/endoplasmic reticulum calcium ATPase (SERCA) pump [258]. ER calcium depletion activates ER stress sensors, including IRE1 and PERK, through dissociation from the chaperone BiP. Activated IRE1 recruits TRAF2 [259], leading to activation of c-Jun N-terminal kinase (JNK) via apoptosis signal-regulating kinase 1 (ASK1) [260]. JNK promotes the formation of c-Fos/Jun-B dimers, which bind to the activator protein-1 (AP-1) binding site of the CHOP (CCAAT/enhancer-binding protein homologous protein) gene, inducing its expression [261]. Concurrently, PERK phosphorylates eIF2α, which activates ATF4 and subsequently upregulates CHOP [262]. CHOP promotes apoptosis by downregulating anti-apoptotic Bcl-2 proteins and upregulating pro-apoptotic members such as Bim [258,262,263,264,265].

Another consequence of ER calcium depletion is elevated cytosolic calcium levels [262,264], which activate calpain-2 [266] and subsequently caspase-12 [239,265,267,268], further contributing to β-cell apoptosis.

### 11.3. Oxidative Stress and Inflammation

Oxidative stress and inflammation are additional contributors to β-cell apoptosis [269,270,271,272,273]. Fatty acid β-oxidation in peroxisomes generates hydrogen peroxide (H_2_O_2_) via electron transfer to oxygen, catalyzed by acyl-CoA oxidase [237,238]. While peroxisomes possess catalase to detoxify H_2_O_2_, β-cells are particularly vulnerable due to the absence of catalase expression in their peroxisomes [274,275,276]. As a result, H_2_O_2_ diffuses into the cytosol, where it is detoxified by glutathione peroxidase [238]. However, rapid H_2_O_2_ accumulation, especially when combined with superoxide radicals to form highly reactive hydroxyl radicals [239], can overwhelm antioxidant defenses, leading to oxidative stress and inflammation [240].

Fatty acid chain length influences cytotoxicity. Long-chain fatty acids (10–16 carbons) are oxidized in both mitochondria and peroxisomes, whereas very long-chain fatty acids (≥17 carbons) are preferentially metabolized in peroxisomes [238,277]. Importantly, only long-chain fatty acids (≥14 carbons) have been shown to be cytotoxic to β-cells [278]. Palmitate, for example, increases H_2_O_2_ production in β-cell peroxisomes, leading to cell death. Overexpression of catalase in either the peroxisome or cytosol mitigates H_2_O_2_ accumulation and prevents palmitate-induced β-cell death [278].

### 11.4. Apoptosis and Type 2 Diabetes Progression

Apoptosis plays a critical role in the progression of type 2 diabetes. β-cell apoptosis is a hallmark of established diabetes in both animal models [279] and humans [56,280]. Consistently, β-cell mass is reduced in individuals with type 2 diabetes [56,281,282], and this decline correlates with disease duration due to increased apoptotic activity [56,283]. Thus, β-cell apoptosis contributes to the worsening of glycemic control over time.

However, it is important to note that β-cell apoptosis may not be a primary cause of type 2 diabetes onset. A study using pancreatic tissue from adult human cadaveric donors found no significant difference in β-cell apoptosis between lean individuals with type 2 diabetes and lean non-diabetic controls [57], suggesting that apoptosis may not be a key initiating factor in the development of the disease.

### 11.5. Temporary Ectopic Triglyceride Accumulation Protects Against Apoptosis

It is important to note that transient ectopic triglyceride accumulation in otherwise healthy tissue may offer protection against lipotoxicity induced by elevated levels of circulating fatty acids [284]. Experimental evidence has shown that increasing the conversion of fatty acids—such as palmitate and oleate—into triglycerides reduces cellular apoptosis triggered by high fatty acid concentrations. Conversely, inhibition of this conversion process enhances apoptosis [285].

## 12. Examples of Reducing Ectopic Triglyceride Deposition in Type 2 Diabetes Remission

### 12.1. Reversal of Type 2 Diabetes by Bariatric Surgery

Bariatric surgery encompasses a range of procedures designed to promote weight loss by reducing stomach capacity and limiting food intake. Common techniques include Roux-en-Y gastric bypass (RYGB) [286], adjustable gastric banding [287], sleeve gastrectomy [288], biliopancreatic diversion with duodenal switch [289], and vertical banded gastroplasty [290]. These interventions are effective in achieving substantial and sustained weight loss in individuals with severe obesity, often resulting in significant improvements in metabolic health [291].

One of the most notable benefits of bariatric surgery is its ability to normalize hyperglycemia in patients with type 2 diabetes. In an observational study, Pories et al. reported that Greenville gastric bypass surgery normalized blood glucose levels in 79% (80 out of 101) of obese patients with type 2 diabetes at one-year follow-up [54]. Factors contributing to non-responsiveness included surgical failure, excessive food intake, older age, longer diabetes duration, and more advanced disease severity. Remarkably, nearly 90% of responders remained free of diabetes a decade later [49]. The effectiveness of bariatric surgery in improving glycemic control is closely linked to the degree of weight loss achieved [54].

The Swedish Obese Subjects Study, a case–controlled investigation, demonstrated significantly higher diabetes remission rates among 641 obese patients who underwent various forms of gastric surgery—including banding, vertical banded gastroplasty, and gastric bypass—compared to 627 matched obese controls receiving conventional treatment over a 10-year follow-up period [292].

In a randomized prospective study involving 60 patients with recently diagnosed type 2 diabetes (<2 years), Dixon et al. [293] found that laparoscopic adjustable gastric banding (LAGB) normalized mean fasting plasma glucose levels, whereas only modest improvements were observed in the intensive medical treatment group. At the two-year follow-up, diabetes remission was achieved in 73% of the surgical group compared to just 13% in the medically treated group [293].

Interestingly, the glucose-lowering effects of bariatric surgery are not consistently accompanied by changes in blood pressure [292,294], LDL-cholesterol [294,295,296], or total cholesterol [293,294,296]. However, an increase in HDL-cholesterol is commonly observed following surgery [292,293,294,295]. Despite this, HDL does not appear to mediate the early glycemic improvements, as blood glucose levels decrease within three months post-surgery, while HDL levels initially decline before returning to baseline at six months and rising significantly by one year, with sustained elevation for at least five years [296]. Similarly, Heffron et al. reported reductions in HbA1c six months after gastric bypass surgery, without concurrent changes in HDL-cholesterol levels during that period [297].

In contrast, the glucose-lowering effect of bariatric surgery is consistently associated with a reduction in plasma triglyceride levels [292,293,294,295,297] (Table 2). This decrease in circulating triglycerides parallels a reduction in ectopic triglyceride deposition in the liver and skeletal muscle [298]. Collectively, current evidence suggests that the improvement in glycemic control following bariatric surgery is primarily mediated by a reduction in ectopic triglyceride accumulation.

**Figure 22:**
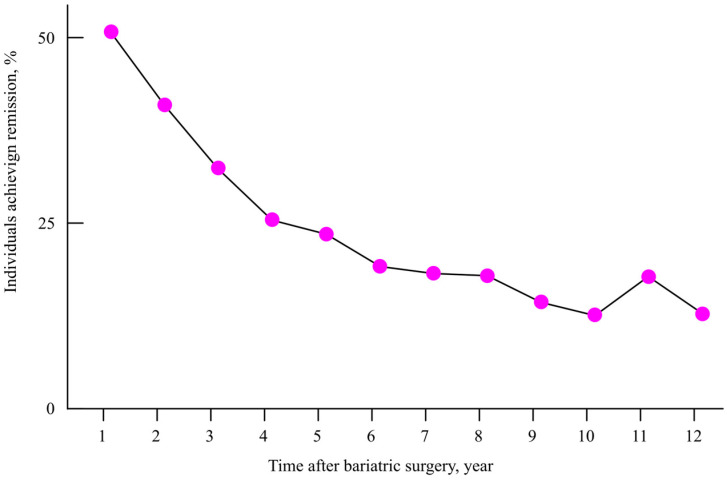
Diabetes remission after bariatric surgery. Remission was defined as hemoglobin A1c < 6.5% and not receiving any medications for diabetes. Data were derived from [294].

While bariatric surgery has demonstrated significant short-term benefits in glycemic control, the remission of type 2 diabetes tends to decline over time. For example, Courcoulas et al. reported a reduction in diabetes remission rates from 51% at one year to just 18% at seven years post-surgery [294] (Figure 22). Despite its effectiveness, bariatric surgery is typically recommended only for individuals with a body mass index (BMI) ≥ 40, or ≥35 in the presence of obesity-related comorbidities [290].

The limited adoption of bariatric surgery is partly due to prevailing perceptions of obesity as a self-inflicted condition resulting from poor lifestyle choices, leading to the view that surgical intervention may be excessively aggressive [299,300,301]. Consequently, fewer than 1% of individuals with a BMI ≥ 35 consider or pursue bariatric surgery as a treatment option [299].

### 12.2. Reversal of Type 2 Diabetes by Dietary Energy Restriction

**Figure 23:**
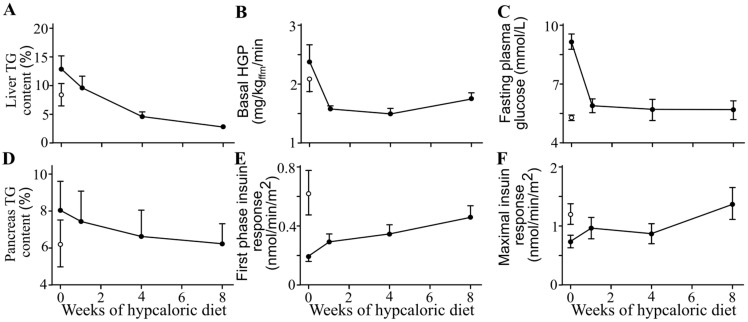
Effect of 8 weeks of dietary energy restriction intervention on (**A**) liver triglyceride (TG) content, (**B**) hepatic glucose production (HGP), (**C**) plasma glucose, (**D**) change in TG content in the pancreas, (**F**) first-phase insulin response, and (**E**) maximal insulin response in diabetic participants (black circles). White circles indicate the mean of the weight-matched non-diabetic control group. Error bar = standard error. Adapted from [59] which was published under the terms of the Creative Commons Attribution Noncommercial License (https://creativecommons.org/licenses/by-nc/4.0/, accessed on 1 July 2025) and from [49] with permission (copyright year, 2013, with permission from John Wiley and Sons).

Dietary energy restriction leads to a rapid and substantial reduction in ectopic triglyceride accumulation in the liver. This reduction is closely associated with the normalization of hepatic insulin sensitivity—evidenced by decreased hepatic glucose production—and a return to normal fasting plasma glucose levels within just seven days [59] (Figure 23). Continued caloric restriction over eight weeks also results in a gradual decline in ectopic triglyceride content in the pancreas, which is accompanied by the restoration of normal insulin secretion [49,59]. These findings strongly support the hypothesis that ectopic triglyceride deposition plays a causal role in the pathogenesis of type 2 diabetes.

## 13. Lowering Triglycerides by Fibrates, but Not Omega-3 Fatty Acids or Niacin, Decreases Insulin Resistance and Protects Against Type 2 Diabetes

Three major classes of medications are commonly used to reduce circulating triglyceride levels: omega-3 fatty acids, niacin, and fibrates [302,303,304]. These agents operate through distinct mechanisms. Omega-3 fatty acids and niacin primarily inhibit triglyceride synthesis [305,306], whereas fibrates enhance fatty acid β-oxidation [307,308].

**Figure 24:**
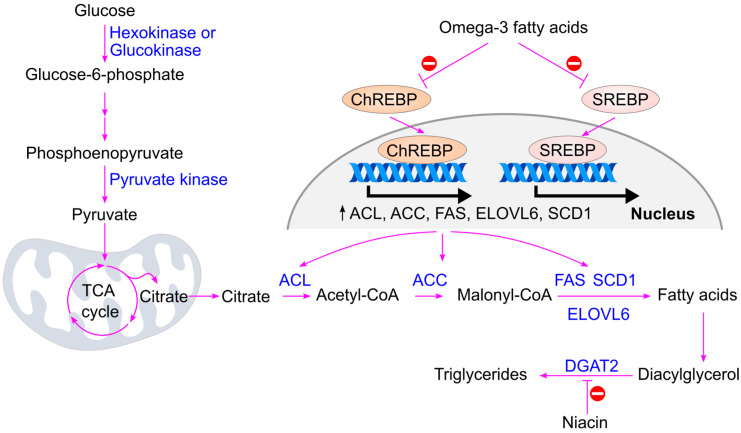
Omega-3 fatty acids and niacin inhibit the conversion of glucose to triglycerides. Omega-3 fatty acids inhibit both ChREBP and SREBP (two transcriptional factors) and thus decrease the expression of genes involved in triglyceride synthesis. Niacin inhibits DGAT2 to prevent triglyceride formation. ↑, increase; ACC, acetyl-CoA carboxylase; ACL, ATP citrate lyase; ChREBP, carbohydrate response element binding protein; DGAT2, diacylglycerol acyltransferase 2; ELOVL6, fatty acid elongase 6; FAS, fatty acid synthase; SCD1, stearoyl-CoA desaturase; SREBP, sterol regulatory element-binding protein. Blue text indicates enzymes.

Omega-3 fatty acids suppress the activity of key lipogenic transcription factors ChREBP and SREBP [134,309] (Figure 24). These transcription factors bind to their respective response elements in the nucleus and upregulate genes involved in fatty acid synthesis, including acetyl-CoA carboxylase, ATP citrate lyase, fatty acid synthase, elongase 6, and stearoyl-CoA desaturase [128,132,133,134]. By inhibiting these pathways, omega-3 fatty acids reduce the conversion of glucose into triglycerides. Niacin, on the other hand, inhibits diacylglycerol acyltransferase 2 (DGAT2), a key enzyme in triglyceride synthesis [306,310,311], thereby reducing triglyceride production [312] (Figure 24).

Because omega-3 fatty acids and niacin reduce triglyceride synthesis, they may limit the capacity of the liver and muscle to convert excess glucose into triglycerides. This could potentially impair glucose disposal and exacerbate hyperglycemia. Indeed, omega-3 fatty acids have not been shown to protect against type 2 diabetes [313], and niacin therapy has been associated with a modestly increased risk of developing diabetes in humans [314,315].

**Figure 25:**
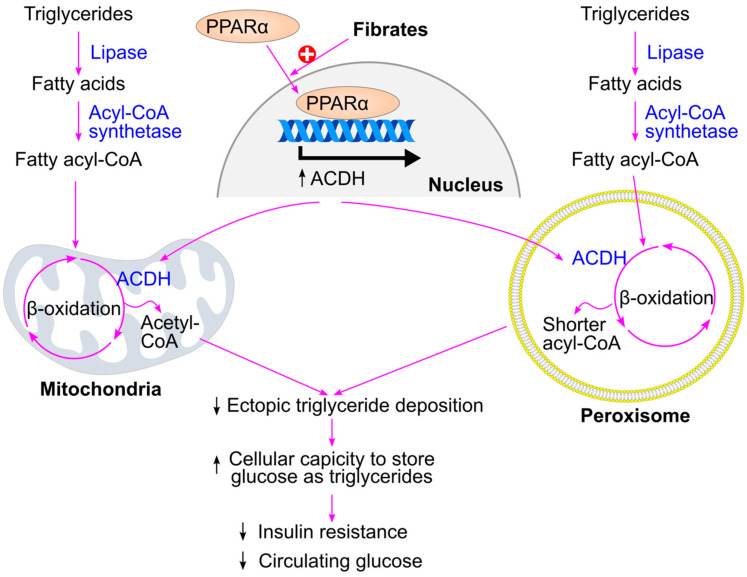
Fibrates decrease ectopic triglyceride deposition and protect against type 2 diabetes. ↓ decrease; ↑, increase; ACDH, acyl-CoA dehydrogenase; PPARα, peroxisome proliferator-activated receptor α. Blue text indicates enzymes.

In contrast, fibrates lower triglyceride levels by promoting fatty acid β-oxidation in mitochondria and peroxisomes (Figure 25). Mechanistically, fibrates activate peroxisome proliferator-activated receptor alpha (PPARα), which induces the expression of genes involved in β-oxidation, such as acyl-CoA dehydrogenase [307,308]. This enhances the metabolic capacity of the liver and muscle to oxidize fatty acids, thereby reducing ectopic triglyceride accumulation and improving glucose handling. As a result, fibrates are expected to confer protective effects against type 2 diabetes. Supporting this, fenofibrate has been shown to reduce insulin resistance and plasma glucose levels [236], protect against diabetes development in animal models [16], and slow the progression of diabetic complications such as albuminuria [18] and retinopathy [19] in humans. Additionally, bezafibrate has been reported to reduce the incidence of type 2 diabetes in clinical studies [17].

Collectively, these findings support the hypothesis that impaired cellular capacity to convert glucose into triglycerides—similar to the pathological accumulation of ectopic triglycerides—is a contributing factor in the development of type 2 diabetes.

## 14. Ectopic Triglyceride Deposition and Cardiometabolic Diseases

Cardiovascular diseases—including coronary artery disease, cerebrovascular disease, peripheral artery disease, and aortic atherosclerosis—remain the leading cause of death worldwide [316,317,318,319,320]. Cardiometabolic diseases encompass a cluster of interrelated conditions, combining metabolic disorders such as obesity and type 2 diabetes with cardiovascular complications like coronary artery disease and heart failure [321]. Elevated triglyceride levels are recognized risk factors for atherosclerosis and its associated cardiovascular events, including myocardial infarction and ischemic stroke [322,323,324], as well as for insulin resistance and diabetes. These associations suggest that ectopic triglyceride deposition may represent a common pathological mechanism underlying cardiometabolic diseases. However, whether it serves as a unified causal factor across these conditions warrants further investigation.

## 15. Triglyceride Paradox in Cardiovascular Disease

Elevated levels of circulating triglycerides are traditionally considered a risk factor for atherosclerotic cardiovascular disease. Triglyceride-rich lipoprotein remnants can become trapped in the subendothelial space, where they promote inflammatory responses and accelerate atherogenesis [325]. Numerous randomized clinical trials have demonstrated the cardiovascular benefits of triglyceride-lowering therapies, including fibrates [326,327,328], niacin [329], and omega-3 fatty acids [330,331], although some studies have failed to show a protective effect [315,332,333].

Paradoxically, several studies have reported that in patients with acute ischemic stroke, higher admission triglyceride levels are inversely associated with stroke severity [334,335,336] and with short-term mortality (within 3 months) [335,336] as well as long-term mortality (within 5 years) [337]. Furthermore, Xia et al. found that admission triglyceride levels were associated with both all-cause and cardiovascular mortality in patients with coronary artery disease [338].

The underlying mechanisms of this triglyceride paradox in cardiovascular disease remain unclear. One hypothesis is that low triglyceride levels may reflect poor overall nutritional status in these patients [335,339]. Additionally, extremely low triglyceride concentrations may impair the synthesis of phospholipids [215], which are essential for maintaining the structural integrity and stability of cell membranes [338,340].

## 16. Clinical Utility of Triglycerides as Biomarkers

Elevated triglyceride levels can serve as a biomarker for adipose tissue dysfunction. Circulating triglycerides have been shown to correlate positively with adipose tissue insulin resistance [341] and with soluble CD163 (sCD163), a marker of macrophage activation within adipose tissue [342,343]. Additionally, triglyceride levels are inversely associated with adiponectin, an adipokine that reflects insulin sensitivity in adipose tissue [344]. Adipose tissue dysfunction contributes to hypertriglyceridemia through increased hepatic production of very-low-density lipoproteins and impaired triglyceride hydrolysis [345].

Triglycerides also have clinical utility as biomarkers for non-alcoholic fatty liver disease (NAFLD). Elevated circulating triglycerides, especially in the context of high dietary fat and sugar intake, promote ectopic triglyceride accumulation in the liver. Numerous studies have documented a positive association between high triglyceride levels and NAFLD [346]. The fatty liver secretes over 20 hepatokines into the circulation, influencing systemic metabolism—a topic recently reviewed in detail [284]. One notable hepatokine is fetuin-A [347], a natural inhibitor of insulin receptor tyrosine kinase activity in both liver and skeletal muscle [348,349].

## 17. Lipoprotein Lipase, Insulin Resistance, and Hypertriglyceridemia

Lipoprotein lipase hydrolyzes circulating triglycerides into fatty acids at the luminal surface of capillaries by binding to glycosylphosphatidylinositol-anchored high-density lipoprotein-binding protein (GPIHBP1) [350]. This enzymatic activity facilitates the uptake of fatty acids by peripheral tissues and contributes to the reduction in circulating triglyceride levels [351,352].

In transgenic mice with muscle-specific overexpression of lipoprotein lipase, intramuscular triglyceride content increased threefold. These mice exhibited insulin resistance, characterized by a reduction in insulin-stimulated glucose uptake in skeletal muscle [353]. Similarly, liver-specific overexpression of lipoprotein lipase led to a twofold increase in hepatic triglyceride content and impaired insulin-mediated suppression of endogenous glucose production, indicating hepatic insulin resistance [353]. These findings support the hypothesis that ectopic triglyceride accumulation in skeletal muscle and liver contributes to the development of insulin resistance.

In patients with type 2 diabetes, both lipoprotein lipase production and circulating lipoprotein lipase levels are reduced [350,354]. Additionally, the levels of angiopoietin-like proteins 3, 4, and 8—known inhibitors of lipoprotein lipase activity [351]—are elevated in these individuals [355,356,357]. Consequently, the clearance of triglyceride-rich lipoproteins is impaired under diabetic conditions, contributing to elevated circulating triglyceride concentrations [357].

## 18. Conclusions

**Figure 26:**
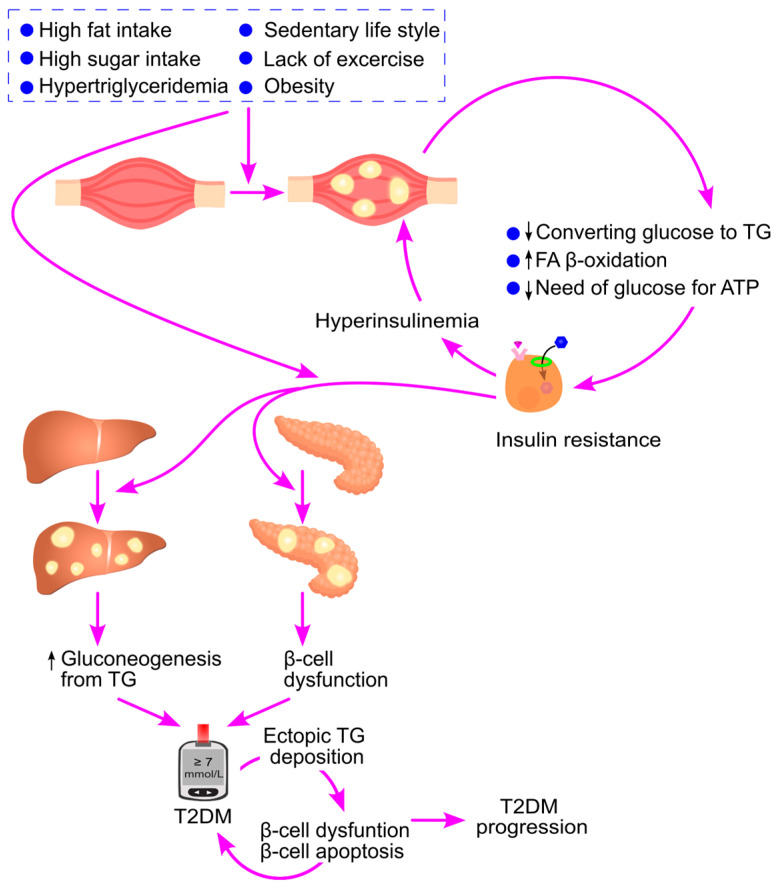
Proposed mechanisms linking ectopic triglyceride deposition to the development and progression of type 2 diabetes. ↓ decrease; ↑, increase; FA, fatty acid; T2DM, type 2 diabetes; TG, triglyceride.

Ectopic triglyceride accumulation can result from poor dietary and lifestyle habits, including excessive intake of fats and sugars, physical inactivity, and a sedentary lifestyle [358]. In addition, obesity and hypertriglyceridemia are key contributors to ectopic fat deposition in non-adipose tissues such as the liver, pancreas, and skeletal muscle (Figure 26). This abnormal lipid accumulation impairs the cellular capacity to convert glucose into triglycerides for storage and simultaneously increases fatty acid oxidation. As a result, cells rely less on glucose for ATP production, contributing to insulin resistance and compensatory hyperinsulinemia. Elevated insulin levels further promote fatty acid uptake and exacerbate ectopic triglyceride deposition [192], creating a self-reinforcing cycle.

Initially, insulin resistance in skeletal muscle may be offset by increased glucose storage as triglycerides in adipose tissue. However, as insulin resistance progresses in adipose tissue, this compensatory mechanism becomes ineffective. The subsequent rise in ectopic triglyceride deposition in the liver and pancreas leads to increased hepatic gluconeogenesis and β-cell dysfunction. Eventually, the β-cell compensatory response fails to maintain normoglycemia, culminating in the onset of type 2 diabetes.

Type 2 diabetes further accelerates ectopic triglyceride deposition. Patients with diabetes exhibit a higher rate of triglyceride accumulation in skeletal muscle from circulating lipids compared to healthy individuals [61]. Moreover, diabetes amplifies the detrimental effects of ectopic fat on β-cell function. In diabetic rats, high-fat feeding impairs glucose-stimulated insulin secretion (GSIS), whereas the same diet has no effect in normoglycemic controls [359]. Hyperglycemia and elevated intracellular levels of triglycerides and fatty acids also contribute to β-cell apoptosis—a hallmark of established type 2 diabetes. Patients with type 2 diabetes typically exhibit a 25–60% reduction in β-cell mass [136,360,361,362], and GSIS capacity declines progressively [1,362,363]. Consequently, approximately 50% of individuals with type 2 diabetes require insulin therapy within 6–10 years of diagnosis [59,60].

Current dietary fat recommendations for individuals with type 2 diabetes, as outlined by the American Diabetes Association, emphasize fat quality over quantity. Specifically, they advocate for dietary patterns rich in monounsaturated and polyunsaturated fats—such as those found in fatty fish, nuts, and seeds—consistent with a Mediterranean-style eating plan, to reduce cardiovascular risk and improve glucose metabolism [364,365]. However, the present review argues that reducing the overall quantity of dietary fat intake may also warrant consideration as a strategy to mitigate ectopic triglyceride deposition and improve metabolic outcomes in type 2 diabetes.

## Figures and Tables

**Table 1:** Examples of common saturated and unsaturated fatty acids.

Name	Carbon Skeleton	Structure
Saturated fatty acids		
Butyric acid	4:0	CH_3_(CH_2_)_2_COOH
Valeric acid	5:0	CH_3_(CH_2_)_3_COOH
Lauric acid	12:0	CH_3_(CH_2_)_10_COOH
Myristic aid	14:0	CH_3_(CH_2_)_12_COOH
Palmitic acid	16:0	CH_3_(CH_2_)_14_COOH
Stearic acid	18:0	CH_3_(CH_2_)_16_COOH
Arachidic acid	20:0	CH_3_(CH_2_)_18_COOH
Lignoceric acid	24:0	CH_3_(CH_2_)_22_COOH
Unsaturated fatty acids		
Palmitoleic acid	16:1 (∆^9^)	CH_3_(CH2)_5_CH=CH(CH_2_)_7_COOH
Oleic acid	18:1 (∆^9^)	CH_3_(CH2)_7_CH=CH(CH_2_)_7_COOH
Linoleic acid	18:2 (∆^9,12^)	CH_3_(CH_2_)_4_CH=CHCH_2_CH=CH(CH_2_)_7_COOH
α-Linolenic acid	18:3 (∆^9,12,15^)	CH_3_CH_2_CH=CHCH_2_CH=CHCH_2_CH=CH(CH_2_)_7_COOH
Arachidonic acid	20:4 (∆^5,8,11,14^)	CH_3_(CH_2_)_4_CH=CHCH_2_CH=CHCH_2_CH=CHCH_2_CH=CH(CH_2_)_3_COOH
EPA	20:5 (∆^5,8,11,14,17^)	CH_3_CH_2_CH=CHCH_2_CH=CHCH_2_CH=CHCH_2_CH=CHCH_2_CH=CH (CH_2_)_3_COOH
DHA	22:6 (∆^4,7,10,13,16,19^)	CH_3_CH_2_CH=CHCH_2_CH=CHCH_2_CH=CHCH_2_CH=CHCH_2_CH=CH CH_2_CH=CH(CH_2_)_2_COOH

DHA, docosahexaenoic acid; EPA: eicosapentaenoic acid.

**Table 2:** Some studies showing that bariatric surgery decreases triglycerides.

Study: Author, Year	Triglyceride Change in the Surgery Group	Triglyceride Change in the Control Group	*p* Value	Reference
Sjöström et al, 2004	−16.3 mg/dL	2.2 mg/dL	<0.001	[292]
Dixon et al, 2008	−71.7 mg/dL	2.1 mg/dL	0.02	[293]
Courcoulas et al, 2024	−19.0%	2.3%	0.002	[294]
Kirwan et al, 2022	−48 mg/dL	−10 mg/dL	0.004	[295]
Heffron et al, 2018	−13 mg/dL	N/A	N/A	[297]

N/A: not applicable.

## Data Availability

No new data were created or analyzed in this study. Data sharing is not applicable to this article.
